# A sophisticated, differentiated Golgi in the ancestor of eukaryotes

**DOI:** 10.1186/s12915-018-0492-9

**Published:** 2018-03-07

**Authors:** Lael D. Barlow, Eva Nývltová, Maria Aguilar, Jan Tachezy, Joel B. Dacks

**Affiliations:** 1 Department of Cell Biology, Faculty of Medicine and Dentistry, University of Alberta, 5-31 Medical Sciences Building, Edmonton, Alberta T6G 2H7 Canada; 2 Department of Parasitology (BIOCEV), Faculty of Science, Charles University, Průmyslová 595, 252 42 Vestec, Czech Republic; 3 Department of Neurology, University of Miami Miller School of Medicine, 1600 NW 10th Avenue, Rosenstiel Medical Science Building (RMSB) # 2067, Miami, Florida, 33136 USA; 4 Department of Life Sciences, The Natural History Museum, Cromwell Road, London, SW7 5BD UK

**Keywords:** Golgi apparatus, GRASP, golgin, evolutionary cell biology, secretion, membrane trafficking

## Abstract

**Background:**

The Golgi apparatus is a central meeting point for the endocytic and exocytic systems in eukaryotic cells, and the organelle’s dysfunction results in human disease. Its characteristic morphology of multiple differentiated compartments organized into stacked flattened cisternae is one of the most recognizable features of modern eukaryotic cells, and yet how this is maintained is not well understood. The Golgi is also an ancient aspect of eukaryotes, but the extent and nature of its complexity in the ancestor of eukaryotes is unclear. Various proteins have roles in organizing the Golgi, chief among them being the golgins.

**Results:**

We address Golgi evolution by analyzing genome sequences from organisms which have lost stacked cisternae as a feature of their Golgi and those that have not. Using genomics and immunomicroscopy, we first identify Golgi in the anaerobic amoeba *Mastigamoeba balamuthi*. We then searched 87 genomes spanning eukaryotic diversity for presence of the most prominent proteins implicated in Golgi structure, focusing on golgins. We show some candidates as animal specific and others as ancestral to eukaryotes.

**Conclusions:**

None of the proteins examined show a phyletic distribution that correlates with the morphology of stacked cisternae, suggesting the possibility of stacking as an emergent property. Strikingly, however, the combination of golgins conserved among diverse eukaryotes allows for the most detailed reconstruction of the organelle to date, showing a sophisticated Golgi with differentiated compartments and trafficking pathways in the common eukaryotic ancestor.

**Electronic supplementary material:**

The online version of this article (10.1186/s12915-018-0492-9) contains supplementary material, which is available to authorized users.

## Background

At the intersection of the secretory and endocytic membrane-trafficking pathways in eukaryotes lies the Golgi. This organelle comprises a series of compartments termed cisternae, providing a platform for protein transport, glycosylation, and targeting. The Golgi is crucially important for normal cellular function, as demonstrated by the myriad diseases that result when genes associated with it are mutated [1]. The most salient hallmark of Golgi structure is the presence of multiple membranous compartments, differentiated into *cis*, *medial*, and *trans*-Golgi, and organized into flattened stacks, which facilitates many key Golgi functions in mammalian cells [2]. In mammalian cells, numerous proteins are involved in maintaining the structure and positioning of the Golgi, as well as the specificity of membrane trafficking pathways to and from the Golgi [3], although the precise mechanism of Golgi stacking is unknown.

Golgins and Golgi reassembly and stacking proteins (GRASPs) are the main factors implicated in Golgi organization and stacking, as reviewed previously [4]. The golgins are a collection of 11 proteins in mammalian cells defined by the presence of coiled-coil domains, attachment to Golgi membranes near their C-termini (either by tail-anchor transmembrane domains or through binding to small GTPases), and functions that include tethering/scaffolding [3, 5]. The domain topology and functions of mammalian golgins have been reviewed extensively elsewhere [3, 6]. Striking evidence for a role of GRASP55, GRASP65, GM130, and golgin-45 in stacking was shown by a knock-sideways experiment demonstrating that ectopic expression of GRASP55 on mitochondria is sufficient for the stacking of mitochondrial and Golgi membranes together [7]. A similar ectopic expression of golgin-84 on mitochondrial membranes also caused stacking of mitochondria [8]. In addition to apparent roles in stacking, golgins, including GM130 and golgin-84, are involved in tethering specific transport vesicles destined for different regions of the Golgi [8]. Furthermore, several golgins, including GM130, are involved in connecting the Golgi to the cytoskeleton [9, 10]. Various additional proteins have also been suggested to be involved in Golgi structure and organization (Additional file 1: Table S1).

The integral role of golgins and other implicated structural proteins at the Golgi makes their evolutionary histories essential to reconstructing both the nature of the Golgi in the last eukaryotic common ancestor (LECA) approximately 1.5 billion years ago [11], and to tracing the subsequent changes that have occurred in the evolution of diverse eukaryotic lineages. While it has been inferred that the LECA possessed a stacked Golgi [12], whether there are pan-eukaryotic proteins (e.g., golgins) that may have conserved roles in Golgi stacking remains unknown. Furthermore, the extent and details of golgin-mediated vesicle trafficking in the diversity of eukaryotes as compared with mammalian cells is also an open question.

Intriguingly, while Golgi stacking is observed in most organisms across eukaryotic diversity, there are a few lineages of microbial eukaryotes that lack stacked Golgi, as reviewed previously [12]. In the absence of a morphologically recognizable Golgi, the question arose, for each of these lineages as to whether the organelle (1) was ever present, (2) was present but is no longer a feature of the cellular configuration, or (3) is present but has been shifted to an unrecognizable morphology.

Phylogenetic analysis to determine the evolutionary relationships of these organisms has placed them as embedded within various different eukaryotic groups, in almost every case having relatives with canonical stacked Golgi, rather than related to other organisms lacking stacks [13–16]. Furthermore, in every case yet examined, when genome-scale data became available, genes were identified that encode orthologues of proteins that function at the Golgi in mammalian and yeast systems [16–19]. Localization data and functional assays have also confirmed that these proteins are expressed and indeed have shown that discrete Golgi, of morphologies other than stacked cisternae, exist in several of these lineages [19–22]. Recent genomic data for diverse eukaryotes, including from additional organisms with evidence for unstacked Golgi, therefore present the opportunity to understanding the evolution of Golgi structure across the broadest span of eukaryotes and organelle morphologies.

Herein, we report an analysis of golgins and other Golgi structure-associated proteins across eukaryotes, using genomics, molecular cell biology, and bioinformatics techniques to address evolutionary cell biology of the Golgi in eukaryotes.

## Results

### The genome of the “Golgi-less” amoeba *M. balamuthi* encodes Golgi proteins

**Fig. 1:**
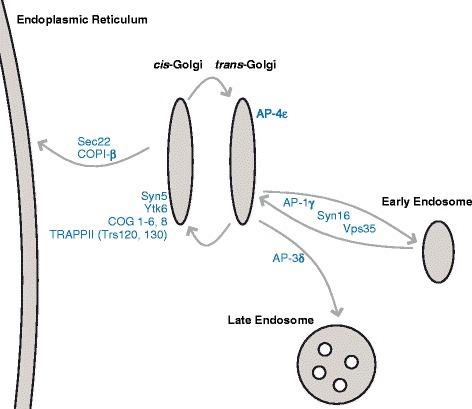
Diagram showing the Golgi marker genes found in *M. balamuthi* and their location in a generalized eukaryotic cell (see Additional file 3: Table S2 for further details). Notably, we identified proteins with roles in vesicle fusion and formation, transport to and from the Golgi, and whose orthologues act at both the *cis* and *trans* faces of the organelle in other eukaryotes. Arrows indicate some membrane trafficking pathways that are reconstructed as likely present in the membrane trafficking system of *M. balamuthi*

Genome sequences exist for 11 microbial eukaryotes with evidence for the presence of a Golgi, but presumably in an unstacked morphology. These organisms are spread throughout the diversity of eukaryotes (Additional file 2: Figure S1), yet in the supergroup Amoebozoa only one genus, the parasitic *Entamoeba*, has an unstacked Golgi, which has been characterized to some extent [22]. *M*. *balamuthi* is a free-living anaerobic amoeba, related to *Entamoeba*, that lacks an identifiable stacked Golgi and that was at one time proposed to be lacking the organelle [23]. To expand our sampling of eukaryotic genomes for this comparative analysis, particularly to increase taxon sampling in the Amoebozoa by adding a non-parasitic representative, we searched within the draft genome of *M. balamuthi* (see Methods) for genes that might indicate the presence of a Golgi. A set of Golgi marker genes has been previously established to have been present in the LECA [24], and also as present in the genomes of organisms that lack Golgi stacking [12, 16–19, 25]. Previously seven such proteins were reported for *M. balamuthi* based on individual gene studies [12, 25]. We were able to expand this list to a total of 22 proteins (Fig. 1; Additional file 3: Table S2), including the soluble *N*-ethylmaleimide-sensitive fusion protein attachment protein receptor (SNARE) proteins Syn5, Syn16, and Sec22, the Retromer complex component Vps35, and the components of the multi-subunit tethering complexes that act at the Golgi, COG and TRAPPII. This list also includes the genes encoding the large subunits of the Adaptin 1, 3, and 4 complexes involved in transport from the *trans-*Golgi network (TGN), and the β-subunit of the coat protein complex I (COPI) involved in intra-Golgi transport and traffic from the Golgi back to the endoplasmic reticulum (ER).

### Golgi-like compartments in *M. balamuthi* are dispersed and punctate

**Fig. 2:**
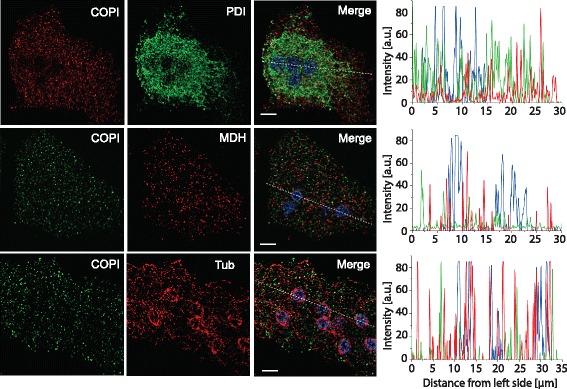
Localization for *M. balamuthi* COPI-β. Structured illumination microscopy of *M. balamuthi* labelled with antibodies against COPI and PDI (top row, ER structure), MDH (middle row, hydrogenosomes), and α tubulin (bottom row). The COPI signal is observed in numerous vesicles scattered within the *M. balamuthi* cells. α tubulin antibody labelled the tubular conus around nuclei and network of fibers. Signal for PDI network is concentrated around multiple nuclei. Graphs show line scans for fluorescence intensities corresponding to the dotted lines in merged images. Scale bar, 5 μm

To validate our genomic and informatics findings, we took a molecular cell biological approach. After further confirming the orthology of the COPI-β orthologue in *M. balamuthi* by phylogenetic analysis (Additional file 4: Figure S2), a specific antibody was raised and validated (Additional file 5: Figure S3), and used for immunofluorescence light microscopy. This showed localization to discrete punctate structures scattered throughout the *M. balamuthi* cytosol, confirming expression of the protein and indicating a vesicular form of the organelle (Fig. 2, bottom row). We did not observe any association of Golgi with cytoskeletal structures of the microtubular conus around the cell’s multiple nuclei and microtubular fibers. We treated *M. balamuthi* with 10 nM, 100 nM, 1 μM, and 10 μM of Brefeldin A for 5 hours and subsequently analyzed the COPI-β signal by SIM. However, we did not observe any difference in comparison to non-treated cells (data not shown). Brefeldin A-insensitive versions of GBF1 (the ArfGEF upon which Brefeldin acts) have been reported in other taxa, such as *Arabidopsis* [26] and *Canis familiaris* [27], and we suggest that this is likely the case here. Consistent with this hypothesis, the relevant amino acid residue for Brefeldin sensitivity in this protein (corresponding to M832 in *Homo sapiens*) is not conserved in *M. balamuthi* (for sequence see Additional file 3: Table S2).

The COPI complex mediates traffic from the Golgi to the ER in eukaryotic cells, and therefore the ER would be a likely location for the COPI complex were a Golgi not present. To ensure that this was not the case, we co-localized the COPI-β with protein disulfide-isomerase (PDI), a well-known ER marker. This showed a PDI signal present in tubular structures close to nuclei as well as in numerous vesicles in the endoplasm, but little overlap with the COPI-β signal (Fig. 2, top row). Furthermore, since hydrogenosomes, the mitochondria-derived organelles in *M. balamuthi*, can also take the form of small discrete punctae [28], co-localization experiments were performed (Fig. 2, middle row) showing no overlap between COPI-β and the hydrogenosomal marker malate dehydrogenase. Together, these informatics and microscopy results are most consistent with the presence of a cryptic unstacked Golgi in *M. balamuthi*, and validate the inclusion of genomic information from this organism in our subsequent searches.

### Evolution of the interacting Golgi structural proteins GM130, golgin-45, GRASP55, and GRASP65

To understand the distribution and evolution of proteins with putative roles in Golgi stacking, we performed comparative genomic searches to assess the taxonomic distribution of mammalian golgins, as well as other Golgi proteins that are either golgin-like (e.g., golgin-45), golgin-associated (e.g., ZFPL1), or GRASPs (Additional file 1: Table S1).

**Fig. 3:**
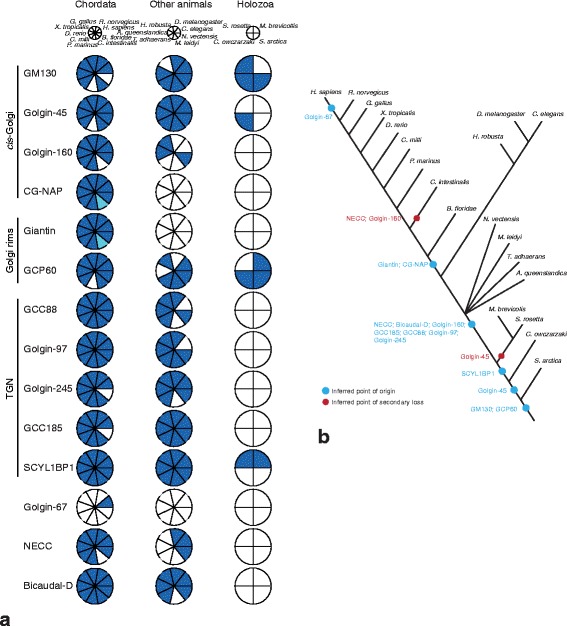
Metazoa-specific golgin evolution. **a** Coulson plot of Metazoa-specific golgin complement. Note that, for this figure and Fig. 4, filled pie sectors represent the positive identification of at least one orthologue (paralogue numbers are not shown). Light blue sectors indicate instances where an orthologue was not found in *Ciona intestinalis* but was found in the genome of a closely related ascidian. This representation is based on data shown in Additional file 2: Figure S1 and Additional file 6: Table S3. **b** Schematic showing timing of gains and losses of metazoan golgin genes. Note that, here and for Fig. 4, gene duplications yielding expanded complements are not tracked and losses are only inferred when a factor was not identified in more than one representative of a taxonomic group

**Fig. 4:**
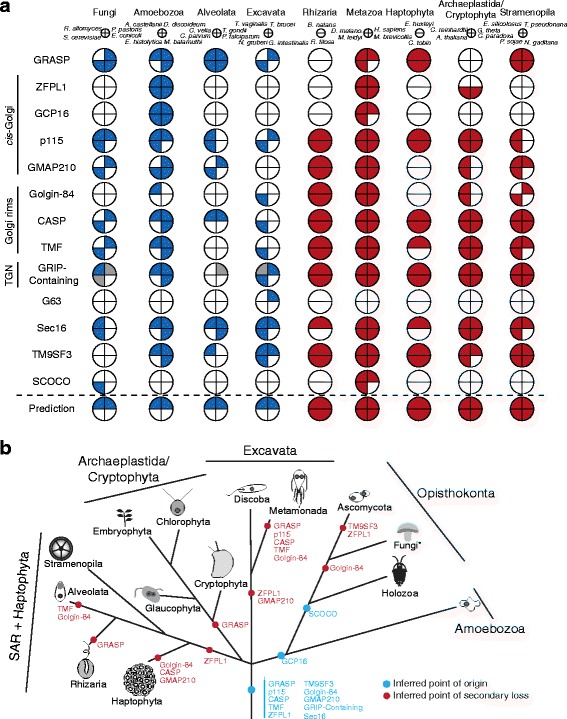
Pan-eukaryotic Golgi protein evolution. **a** Coulson plot of Golgi proteins found outside the Metazoa. Most importantly, while these represent ancient proteins, none show the phylogenetic pattern that would be expected for a necessary stacking factor, illustrated in the “Prediction” row. To clarify the patterns of presence and absence in organisms with stacked and unstacked cisternae, only selected genomes are shown here. The full data are given in Additional file 2: Figure S1 and Additional file 6: Table S3. The first four columns (blue) show genes identified in organisms with unstacked Golgi, and closely related organisms with stacked Golgi, while remaining columns (red) indicate genes identified in representatives of taxonomic groups with stacked Golgi. Gray sectors indicate sequences identified using alternative methods (Additional file 2: Figure S1). **b** Schematic showing the timing of gains and losses of the proteins across eukaryotic evolution. Note that, if a single member of the taxonomic group possesses an orthologue of the protein, it is inferred as present in that group. Relationships between eukaryotes are based on recent concatenated phylogenetic results [75, 101]. To highlight losses in the Ascomycota, they are broken out to the exclusion of the paraphyletic remaining Fungi (denoted by the asterisk)

GM130, golgin-45, GRASP55, and GRASP65 play key roles in Golgi stacking in mammalian cells [4, 7]. GM130 binds to GRASP65 at the *cis-*Golgi, while golgin-45 binds to GRASP55 at the *medial-*Golgi cisternae of mammalian cells [29, 30]. Searches for GM130 and golgin-45 (Fig. 3a; Additional file 2: Figure S1; Additional file 6: Table S3) revealed no homologues outside of animals and their single-celled relatives (Holozoa). Consistent with previous efforts, our analysis did not identify the GM130 analogue Bug1p as a homologue of GM130 in *Saccharomyces* based on sequence similarity [31]. Homologues of GRASP55 and GRASP65 have been previously identified in diverse eukaryotes and functionally studied in organisms both with canonical stacked Golgi [32] and with unusual morphologies [21]. Consistent with this result, and expanding upon it, we found that the duplication into GRASP55 and GRASP65 is a metazoan trait, predating the evolution of jawed fish (Additional file 7: Figure S4), which means that all GRASP proteins in other eukaryotes are pre-duplicates of these two proteins. Also consistent with previous analyses [24, 33], GRASP was found across eukaryotes (Fig. 4a, Additional file 2: Figure S1, and Additional file 6: Table S3) implying its presence in the LECA. However, GRASP was not identified in many cases, most prominently in Embryophyta as previously noted [33] and extended here to the entire clade of Archaeplastida plus Cryptophyta, as well as Rhizaria and Metamonada (Fig. 4).

The above observations suggest that the origin of both GM130 and golgin-45 predates the duplication that produced separate GRASP55 and GRASP65 paralogues, rather than coordinately appearing with them. Recent structural studies have elucidated the interaction between GRASP65 and GM130 [34], and between GRASP55 and golgin-45 [35], suggesting that these binding interactions involve specific residues near the C-terminus of GM130 and golgin-45 interacting with specific residues of GRASP65 and GRASP55, respectively. Evaluation of the conservation of these residues in vertebrates and non-vertebrate holozoan GM130 homologues reveals that residues near the C-termini that are important for binding to GRASP65 are contained in an extended region acquired in a vertebrate ancestor (Additional file 8: Figure S5A). These residues include F975 and I990 of the human orthologue, which have been experimentally shown to be important for binding of GM130 to GRASP65 [34]. GRASP65 may have become specialized for interaction with GM130 in vertebrates through corresponding amino acid substitutions. For example, M164 of GRASP65 is one of several residues that form a hydrophobic cleft occupied by the C-terminus of GM130 [34]. However, while GRASP65 orthologues have either methionine or leucine residues at the position corresponding to M164, GRASP55 orthologues and pre-duplicate GRASP have tyrosine or phenylalanine residues (Additional file 8: Figure S5B). Understanding whether GM130 interacts with preduplicate GRASP proteins in non-vertebrate metazoans will be an important point to resolve to understand both the evolution of Golgi and biology in species of ecological and agricultural importance.

### Evolution of *cis-*Golgi golgins

The *cis*-Golgi receives material through anterograde vesicle transport from the ER and in a retrograde fashion from the *medial*-Golgi and *trans-*Golgi/TGN. Multiple golgins are involved in tethering incoming vesicles at *cis*-Golgi cisternae. Although GM130 is Holozoa specific, one of its interactors, ZFPL1 [36], is more widely conserved and likely present in the LECA (Fig. 4a), consistent with previous identification of a homologue in *Arabidopsis*, which localizes to the *cis*-Golgi [37]. Similar to GM130, golgin-160 appears restricted to Metazoa, and was present in the earliest metazoans, despite being absent in *Drosophila* and *Caenorhabditis* (Fig. 3a). By contrast, its binding partner GCP16 appears to be a more ancient invention, being found in opisthokonts and Amoebozoa (Fig. 4). Even more ancient still are p115 and GMAP210, the homologues of which are found across the diversity of eukaryotes and thus were likely present in the LECA.

Mammalian GMAP210 contains an N-terminal amphipathic alpha helix (ALPS domain), which is important for tethering ER-derived vesicles to the *cis*-Golgi [38]. Using the HeliQuest web service [39], we did not identify any such helices in the first 80 residues of GMAP210 sequences from non-vertebrates, suggesting that this is a lineage-specific mechanism for recognition of vesicles by GMAP210, consistent with previous observations [40]. Additionally, GMAP210 orthologues from non-holozoans do not share the N-terminal tryptophan-containing motif also shown to be involved in recognizing vesicles for tethering to the *cis-*Golgi [40] (Additional file 8: Figure S5C). This motif was previously shown to be necessary for tethering vesicles containing GalNAc-T2 and giantin, but not those containing golgin-84 instead [40], which may indicate lineage-specific trafficking mechanisms as giantin is specific to chordates (Fig. 3b). Increased complexity of GMAP210-mediated trafficking pathways may be due to the presence of an ER–Golgi intermediate compartment (ERGIC) in metazoan cells, as GMAP210 has been shown to be involved in trafficking to both ERGIC and the *cis*-Golgi [41]. In contrast to the N-terminal motifs, the Arf-binding GRAB domain of GMAP210 [42] is conserved in orthologues across eukaryotes (Additional file 8: Figure S5D).

### Evolution of cisternal rim golgins

At least four golgins localize to the rims of Golgi cisternae (including *medial-*Golgi cisternae) in mammalian cells, namely golgin-84, CASP, TMF, and giantin. TMF and golgin-84 have direct roles in vesicle tethering, while giantin appears to be important for organizing Golgi cisternae [43]. Giantin is the most recently evolved, appearing in the chordates (Fig. 3). In contrast to previous suggestions that the *Drosophila* protein lava lamp is a giantin homologue [44], no homologues of giantin were identified in *Drosophila*. However, the origin of the giantin-interacting protein GCP60 (ACBD3) [45] (Additional file 1: Table S1) predates that of giantin, having originated prior to the common ancestor of extant holozoans. Both CASP and golgin-84, however, appear to have been present in the LECA as they can be identified in taxonomically diverse eukaryotic genomes (Fig. 4a and Additional file 2: Figure S1). While golgin-84 and CASP have been identified previously in plants [46, 47], we also identify orthologues of golgin-84 in Excavata, rhizarians, amoebozoans, and a basal opisthokont, and identify CASP in even more numerous taxa (Fig. 4 and Additional file 2: Figure S1).

Golgin-84, CASP, and giantin are anchored to the Golgi rims by transmembrane domains of similar length that share sequence similarity, even among mammalian and plant homologues [48]. Mutation of a conserved tyrosine in the transmembrane domain (TMD) of mammalian CASP prevents export from the ER, suggesting a similar importance for this residue in the TMDs of golgin-84 and giantin [48]. In addition, residues within 100 residues immediately upstream of the TMD of mammalian golgin-84 and giantin, although dissimilar to each other, were shown to be involved in localization of these proteins to the Golgi [49]. The TMD and 100 residues on the cytoplasmic side are sufficient for Golgi localization of the *Arabidopsis* orthologues of both golgin-84 [47] and CASP [46]. Here, we confirm that the TMD and upstream cytoplasmic region of CASP and golgin-84 orthologues are conserved across eukaryotes, including Excavata (Additional file 8: Figure S5E). These observations are consistent with conserved mechanisms of localization of golgin-84 and CASP within the Golgi, which would also have occurred in the LECA’s Golgi.

Mammalian golgin-84 and TMF have previously been shown to contain tryptophan-containing N-terminal motifs similar to that of GMAP210 [40]. Like GMAP210, TMF does not show conservation of this motif outside of metazoans. In contrast, golgin-84 orthologues across eukaryotes contain comparable N-terminal motifs (Additional file 8: Figure S5F). TMF shows conservation within the coiled-coil region that is thought to function in vesicle capture [40] (Additional file 9), as well as its C-terminal Rab6-binding domain [50] (Additional file 8: Figure S5G).

### Evolution of *trans-*Golgi/TGN golgins

Mammalian GRIP (Golgin-97, RanBP2alpha, Imh1p, and P230/golgin-245) domain-containing golgins at the *trans-*Golgi/TGN receive vesicles from various endosomal sources (GCC88, golgin-97, and golgin-245) [8, 51]. The presence of four distinct GRIP golgins in mammalian cells suggests that there might be multiple ancient GRIP golgin paralogues; however, this is not what we observe. All four of the human GRIP golgins (the vesicle tethers and GCC185) appear to be restricted to metazoa (Fig. 3). Non-mammalian GRIP domain-containing proteins include the previously identified and characterized golgins *Saccharomyces* Imh1p [52], *Arabidopsis* AtGRIP [53], and *Trypanosoma* TbGRIP [54]. Herein, GRIP domain-containing proteins are found across all supergroups (Fig. 4a and Additional file 2: Figure S1).

Further, the coiled-coil domain-containing protein SCY1-like 1 binding protein 1 (SCYL1BP1) binds Rab6 at the *trans*-Golgi in mammalian cells, but has unknown function [55]. The origin of SCYL1BP1 predates that of the choanoflagellate lineage of Holozoa (Fig. 3). A potential *Arabidopsis* homologue has been noted previously [56]. This protein was identified but did not meet the criteria for inclusion, whereas proteins that met the E-value cutoffs were identified here in *Guillardia* and *Bigelowiella* (Additional file 6: Table S3). Nevertheless, whether these are true homologues remains ambiguous considering the short length of similar sequence regions as well as the numerous independent gene losses implied by such a patchy distribution of homologues. Should these be true orthologues, then SCYL1BP1 would be deduced to have a much earlier evolutionary origin than stated. However, we suggest that conclusions regarding homology be reserved until functional characterization is available.

### Evolution of additional proteins implicated in Golgi structure

Three golgin-like proteins with functions that have not been assigned to specific Golgi regions were also included in the analysis, and appear to have originated within the Holozoa or Opisthokonta. First, CG-NAP, a protein with function at both the Golgi and the centrosome [57] (Additional file 1: Table S1), originated prior to the divergence of *Branchiostoma* from other chordates. Second, homologues of NECC1/NECC2 were found to have an earlier origin, with identification of a homologue in *Nematostella*, indicating that the origin possibly predated the diversification of the deepest-branching animal lineages (Fig. 3). Third, SCOCO, an Arl1/Arl3-binding protein of unknown function [58, 59], appears to be opisthokont specific, with homologues only identified in fungi and Holozoa (Fig. 4 and Additional file 2: Figure S1).

Finally, an additional three proteins of interest are relevant to the evolutionary investigation of Golgi structure. First, the existence of metazoan-specific golgins suggested that lineage-specific golgin-like proteins may be present in other eukaryotic lineages as well. One such protein has already been identified in kinetoplastids, and the homologue in *Trypanosoma brucei* (TbG63) has been implicated in Golgi organization [60]. Our analyses found that this protein is present in the genome of *Bodo saltans*, the sister lineage to trypanosomatids, but not in any non-kinetoplastids (Additional file 2: Figure S1). Second, although not localized to the Golgi, Sec16 has been shown to be widely conserved [61] and important for Golgi stacking in the yeast *Pichia pastoris*, through its function in regulating COPII coat components at tER exit sites [62, 63]. We recapitulate this finding, albeit with increased sampling. Finally, TM9SF3 is one of four widely conserved TM9 superfamily proteins (or nonaspanins) [64]. It is not orthologous to EMP70 in *Saccharomyces*, which is instead more similar to human TM9SF4. Based on its exclusive Golgi localization and its loss of expression correlated with Golgi fragmentation in mammalian spermatids, TM9SF3 has been implicated in Golgi structure [65]. Our analyses demonstrated that TM9SF3 is found across the span of eukaryotes though not in several taxonomically coherent groups, including ascomycete and basidiomycete fungi, ciliates, and apicomplexans (Fig. 4 and Additional file 2: Figure S1).

## Discussion

By applying comparative information from a broad diversity of eukaryotic organisms, evolutionary cell biology has the potential to provide complementary context to more traditional molecular cell biological studies. We have applied this approach to the evolution and cell biology of the Golgi.

### *M. balamuthi* contains a cryptic Golgi

*M. balamuthi* was one of the organisms originally proposed to lack a Golgi, consistent with the idea at the time that it had diverged prior to the evolutionary emergence of the organelle [23]. This idea of primitive Golgi absence has been fully disproven [25], and ultrastructural work has identified compartments proposed as candidate unstacked Golgi cisternae in some *Mastigamoeba* species (*M. balamuthi* was not imaged) [66]. Nevertheless, the possibility of complete absence of this organelle in any given organism remains viable, as was recently demonstrated for mitochondria [16]. Our genomic and immunomicroscopy data suggests that *M. balamuthi* possesses a cryptic Golgi, possibly composed of distributed vesicles. The precise form and dynamics of the organelle remain interesting open questions, ones that must await the technological development of better tools for molecular cell biology in this organism.

### Holozoa-specific golgins reflect lineage-specific increases in trafficking complexity

Our comparative analyses identified a set of Golgi proteins that appear to have originated within Holozoa and which may reflect increased complexity of both vesicle traffic at the Golgi and connection to the cytoskeleton, relative to a pre-holozoan ancestor. N-terminal vesicle recognition motifs present in mammalian orthologues of GMAP210, TMF, and GRIP golgins, but absent outside of Holozoa, suggest a potential gain of tethering functions in these proteins relative to the ancestral sequences. Additionally, several of the proteins originating within Holozoa, for which functional information is available, have roles in tethering the Golgi to the cytoskeleton, including golgin-160 [67], GM130 [10], GCC185 [68], CG-NAP [10], and bicaudal-D [69]. Cytoskeleton-dependent Golgi positioning along microtubules is important for cellular functions that are essential to metazoan multicellularity, including wound healing [70]. This may explain the relatively recent origin of some of these factors. Despite animal-specific gains in complexity, other eukaryotes may also exhibit comparably complex Golgi. One possibility is that proteins, such as TbG63 as well as undiscovered Golgi proteins in other eukaryotic lineages, reflect parallel increases in complexity, which cannot be inferred by characterization of homologues of human Golgi proteins.

### Conservation of golgins suggests differentiated Golgi compartments were present in the LECA

Counter to the intuitive idea that the ancient ancestor of eukaryotes was simple, molecular evolutionary reconstruction of the LECA has revealed a complement of cell biological machinery that is consistent with a highly complex cell. This applies not only to membrane-trafficking proteins but also to nuclear proteins, the cytoskeleton, mitochondria, and metabolism [71]. The set of pan-eukaryotic Golgi-structural proteins that can be deemed as ancient, which we identify here, adds to this ancestral complexity. This has important implications for the complexity and organization of the Golgi in diverse eukaryotes and in the LECA. The presence of proteins such as p115 and ZFPL1 in non-metazoan eukaryotes raises important questions about Golgi function to be explored in those organisms, given that known binding partners of those proteins are metazoa specific. Evolutionarily, although homologues of p115, GMAP210, golgin-84, CASP, TMF, ZFPL1, and GRIP-containing golgins have been previously identified and localized in plant cells [37, 46, 47, 72], identification of homologues in the extensive taxonomic sampling used here confirms that these were present in the LECA for two reasons. First, it makes the possibility of lateral gene transfer even less likely. Second, identification of CASP, golgin-84, TMF, p115, and TM9SF3 in excavates (*Naegleria gruberi* in particular) provides evidence that they were present in the LECA regardless of uncertainty in the rooting of the eukaryotic tree [73–75].

**Fig. 5:**
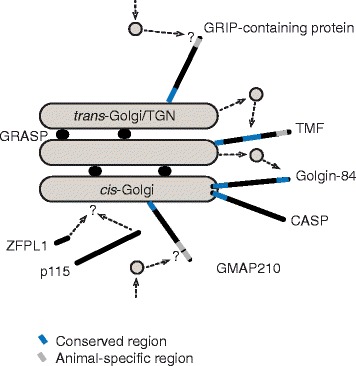
Golgi structure proteins inferred to be present in the LECA. Functional domains and motifs conserved in animals or conserved in the LECA are color coded as inset, and inferred membrane trafficking pathways are shown. Other Golgi proteins were also identified as present in the LECA: TM9SF3 and Sec16. However, their role, if any, in differentiating separate Golgi compartments is unknown

Based on the data collected in metazoan model organisms, and assuming functional homology, the presence of at least four factors at the *cis*-Golgi (p115, GRASP, ZFPL1, and GMAP210) and three at the Golgi rims of successively later cisternae (golgin-84, CASP, and TMF) suggests that the Golgi had differentiated into at least three regions (Fig. 5). Additionally, the conservation of specific sequence motifs provides further evidence for this. The presence of Sec16, which is involved in vesicle formation at ER exit sites, and GMAP210, which receives vesicles from the ER, together with the well-established ancient nature of the COPII coat [61], provides detail of the anterograde trafficking pathways coming into the *cis*-Golgi (Fig. 5). Conservation of the Arf binding GRAB domain in GMAP210 (Additional file 8: Figure S5D) and the previously identified conservation of Arf in eukaryotes, including representatives of Excavata [76], and localization of GMAP210 to the Golgi in Arabidopsis [47] are consistent with conservation of GMAP210 function from the LECA. Tryptophan-containing N-terminal motifs in golgin-84 orthologues from across eukaryotes and in key residues in its transmembrane domain suggest a widely conserved role in intra-Golgi vesicle traffic to the Golgi rims. Similarly, conservation of likely vesicle tethering motifs in TMF suggests a vesicle tethering role for TMF at rims of cisternae closer to the *trans-*Golgi. Again, conservation of Rab6 [77] and the Rab6 binding domain of TMF are also consistent with this (Additional file 8: Figure S5G).

With respect to established TGN compartments, the only inferred LECA golgin at the TGN is a GRIP domain-containing golgin, which acts to receive vesicles from endosomes. The presence of a GRIP domain in proteins across eukaryotic diversity, and the localization of these GRIP domain-containing proteins at the TGN in yeast, plants, and trypanosomes [52, 54, 72] suggests some conserved TGN function from the LECA. The previously identified conservation of Arl1 in eukaryotes, including the representatives of the Excavata, is consistent with conserved function of GRIP golgins [76]. However, the lack of clear conservation of multiple TGN golgins suggests that vesicle traffic to the *trans*-Golgi in non-metazoan cells, and in the LECA, involves fewer specialized tethers and possibly fewer types of transport vesicles. This could also be reflective of the variation of TGN organelles across eukaryotes.

Previous reconstruction of trafficking pathways as present in the LECA, for example, via analysis of COPI, COPII, Retromer, and AP1,4 complexes, as well as Golgi-specific SNARE proteins [78, 79], had suggested potential differentiation of Golgi compartments to some degree. However, these did not indicate whether the ancestral Golgi was a single compartment with specialized domains or was composed of differentiated cisternae. The presence of at least eight ancient proteins implicated in Golgi structure at *cis*-Golgi, cisternal rims, or *trans-*Golgi/TGN, along with conservation of several functional motifs that mediate interactions with binding partners (e.g., Rab6, Arl1, Arf) also reconstructed as present in the LECA, shows that the LECA Golgi was much more complicated than it has been previously possible to infer (Fig. 5). Conservation of golgin-84 and TMF is particularly relevant, as they are specific to intra-Golgi vesicle traffic, which would arguably be unnecessary if Golgi cisternae were not differentiated.

### Golgi stacking is likely an ancient, emergent property

Our analyses also speak to the cell biological question of how Golgi stacking takes place today which, despite its importance and apparent conservation of the stacked morphology of the organelle, remains a matter of significant debate [2]. The predominant paradigm is that one or more Golgi-localized proteins are necessary for the morphology. Given the presence of Golgi stacking across eukaryotes, such a protein could well be predicted to be universal. However, it is not known which proteins, if any, may be necessary for a conserved pan-eukaryotic mechanism of stacking.

By contrast with this paradigm, other suggestions have been put forward to explain Golgi stacking as a morphological property based on several combined factors. This idea has most explicitly been laid out by the “cisternal adhesion” model of Lee et al. [7], whereby one or more proteins with adhesive functions have a stacking effect when present in sufficient quantities. Stacking could also involve regulation of membrane flux through the Golgi, with insufficient input or replenishment as compared to output, causing dissolution of stacks [80]. A model of additive effects of redundant proteins or membrane flux is also consistent with the phenotypes observed in knockouts of retromer components that result in depleted retrograde trafficking from the endosomes to the TGN and fragmentation of the Golgi [81, 82]. The idea that properties of organelles, including Golgi stacking, are dependent on systems-level properties is gaining traction as a viable alternative to exclusively genetic explanations [83]. We collectively denote these hypotheses as Golgi stacking being an emergent property. Overall, the question of how the hallmark morphology of the organelle is established and maintained remains open to debate.

Under the paradigm of a protein with a conserved necessary function in Golgi stacking, such a protein would likely be present in all genomes of organisms showing Golgi stacking, and likely absent from the genomes of those organisms without (i.e., the taxonomic distribution of stacking factors should match that of Golgi stacking). Such a pattern of presence directly correlating with function has been observed for protein complexes responsible for cristae formation in mitochondria [84], and this phylogenetic screening approach has successfully identified proteins involved in flagellar function [85, 86]. The evolutionary analyses performed here across 75 taxa with stacked Golgi and 12 without showed that none of the 27 putative stacking factors that we examined matched this pattern.

There are several caveats to our results. First, individual false positives, or false negatives, are always possible in comparative genomic analyses. Nonetheless, we have used the most accurate homology searching methods, examined datasets of alternate protein models for genomes when relevant and have manually curated the gene assignments. Second, it is conceivable that a universal and necessary stacking gene could exist that possesses multiple functions and so had lost the relevant Golgi function in organisms with unstacked Golgi. However, the fact that every candidate protein examined was apparently absent in multiple genomes of organisms that possess Golgi stacks renders this possibility incompatible with our observations. Finally, it is possible that an as-yet unreported, necessary stacking factor protein may exist, for which we did not search. Proteomics technology allowing distinction between the proteomes of organelles with similar densities, such as the plant ER and Golgi, and even the unique proteomes of organelle sub-compartments [87] may identify previously uncharacterized Golgi proteins that could be candidates for such a necessary stacking factor.

However, accepting these caveats, our results are inconsistent with the hypothesis that any one of the proteins participates in a pan-eukaryotic mechanism of Golgi stacking; this does not discount the importance of lineage-specific functions. Nonetheless, our data are most consistent with Golgi stacking being dependent on an additive, redundant function of non-homologous proteins, i.e., the emergent property hypotheses. An emergent property could rely on ancient redundant proteins, or could rely upon recently evolved, lineage-specific ones that replace ancient factors. With 14 recently evolved proteins identified within the Holozoa (Fig. 3), it is tempting to speculate that additional lineage-specific proteins are also present in other eukaryotes and may have stacking functions. The presence of a kinetoplastid-specific protein (TbG63) is consistent with this scenario, and searches for lineage-specific membrane-trafficking factors associated with clathrin-mediated endocytosis [88] and the sortilin system [89] have certainly been fruitful and illuminating. This will be exciting to pursue in order to understand the mechanisms of Golgi trafficking and stacking, particularly as more genetic and molecular biological tools become available for non-opisthokont model organisms.

Overall, our data do not rule out the existence of a widely conserved necessary stacking factor, but rather support the idea that Golgi stacking as an emergent property needs to be more extensively explored. This may well be the key to understanding one of the most prominent eukaryotic cellular features.

## Conclusions

The cisternal stacking of the Golgi and the separation into *cis-*, *medial-* and *trans-*Golgi compartments is one of the most recognizable aspects of the eukaryotic cell. Our results have allowed insight into both the underlying cell biology and evolution of this prominent eukaryotic feature. At least 10 proteins implicated in Golgi structure have been reconstructed as ancient factors contributing to a differentiated Golgi organelle in the ancestor of eukaryotes over a billion years ago.

## Methods

### Cell cultivation

*M. balamuthi* strain (ATCC 30984) was maintained axenically in PYGC medium at 24 °C in 50 mL culture tissue flask [90]. For immunofluorescence microscopy, *M. balamuthi* cells were fixed in 1% formaldehyde for 30 min, washed, and treated in 1% Triton TX-100 for 10 min. Fixed cells were stained using polyclonal rat anti COPI-β subunit, rabbit anti PDI, rabbit anti MDH [91] Abs, and monoclonal mouse α tubulin (Sigma) Ab. Alexa Fluor 488 (or 594) donkey anti rabbit, Alexa Fluor 594 (or 488) donkey anti rat, and Alexa Fluor 594 donkey anti mouse Abs (Life Technologies) were used as secondary antibodies. Structured illumination microscopy (SIM) was performed using a commercial 3D N-SIM microscope (inverted Nikon Eclipse Ti-E, Nikon) equipped with a Nikon CFI SR Apo TIRF objective (100× oil, NA 1.49). A structured illumination pattern projected into the sample plane was created on a diffraction grating block (100 EX V-R 3D-SIM) for laser wavelengths 488 and 561 nm. Excitation and emission light was separated by filter cubes with appropriate filter sets SIM488 (ex. 470–490, em. 500–545), and SIM561 (556–566, 570–640). Emission light was projected through a 2.5× relay lens onto the chip of an EM CCD camera (AndoriXon Ultra DU897, 10 MHz at 14-bit, 512 × 512 pixels). Three-color z-stacks (z-step: 120 nm) were acquired in NIS-Elements AR software (Laboratory Imaging). Laser intensity, EM gain, and camera exposure time were set independently for each excitation wavelength. The intensity of fluorescence signal was held within the linear range of the camera. Fifteen images (three rotations and five phase shifts) were recorded for every plane and color. SIM data were processed in NIS-Elements AR. Before sample measurement, the symmetry of point spread function was checked with 100 nm red fluorescent beads (580/605, carboxylate-modified microspheres, Life Technologies) mounted in Prolong Diamond Antiface Mountant (Life Technologies), and optimized by adjusting objective correction collar. The signal for 4,6-diamidine-2-phenylindole dihydrochloride (DAPI) was observed in wide-field mode.

### Preparation of antibodies

To obtain complete and partial recombinant PDI and COPI-β proteins, respectively, the corresponding gene sequences were amplified by PCR (Primers: COPI-β forward: CATATGAAGAACCTCGAGCACAGG, COPI-β reverse: AAGCTTCGCGTCGGCCTTGA; PDI forward: CATATGAAGTGGCAGTACATCG, PDI reverse: AAGCTTGAGCTCCTTCTTCTCCCC) using *M. balamuthi* cDNA as template. The PCR products were subcloned into the pET42b+ vector (Novagen), and expressed with a 6xHis tag in *Escherichia coli* BL21 (DE3). The proteins were purified by affinity chromatography under denaturing conditions according to the manufacturer’s protocol (Qiagen) and used to immunize rats (COPI-β) or rabbits (PDI).

### Similarity searches

The genomic databases used for bioinformatics searches are listed in Additional file 10: Table S4. Of note, both the filtered and unfiltered gene model databases at JGI were searched (unfiltered datasets include any redundant gene models for the same gene loci). Additionally, the draft genome of *M. balamuthi*, produced as part of an ongoing project, was searched for conserved Golgi marker and putative stacking factor genes. The draft genome sequence is available at http://www.ebi.ac.uk/ena/data/view/CBKX00000000 (deposited January 22, 2015). The identified gene sequences are detailed and made available in Additional file 3: Table S2.

Basic Local Alignment Search Tool (BLAST 2.2.29+) [92] was used to search for homologues of proteins of interest in *M. balamuthi*-predicted proteins. A bidirectional best-hit criterion was applied with an E-value cut-off of 0.05 for both forward and reverse searches. Additionally, identified sequences were required to retrieve the original query in the reverse search with an E-value of at least two orders of magnitude lower than other sequences. Initial queries are either from the *H. sapiens* or *S. cerevisiae* genomes, or are from other eukaryotes as identified in previous studies [81, 93–95], and multiple queries were used.

For searches to identify orthologues of Golgi structure-associated proteins of interest, a multi-phase approach was taken. BLAST was run locally to search protein sequence databases from a large sampling of eukaryotes (Additional file 10: Table S4). To identify highly similar homologues, reciprocal best hit BLASTP searches were performed using *H. sapiens* query sequences and with the following criteria: E-value of 1 × 10^–20^ or lower for forward search, E-value of 0.05 or lower for reverse search, and a minimum E-value difference of two orders of magnitude, in the reverse BLAST results, between the hit(s) corresponding to the original query and the first negative hit.

HMMER 3.1b1 was then used to perform searches in the same protein sequence databases (http://hmmer.org) [96]. For this, positive hits from BLAST searches were used to build initial Hidden Markov Models (HMMs). Sequences were aligned using MUSCLE v3.8.31 [97] with default parameters. For these searches, the following criteria were applied to define positive hits: E-value of 1 × 10^–10^ or lower for forward (HMMer) search and E-value of 0.05 or lower for reverse (BLASTP) search. After each HMMer search, positive hits, if identified, were aligned and viewed manually before inclusion in HMMs for subsequent searches. This process was repeated until no more positive hits were identified. An exception to these methods was made in the case of the GRIP domain-containing proteins in taxa outside of Metazoa, which were identified using HMMs including only the subsequence of proteins corresponding to the GRIP domain, because no proteins with sequence similarity to individual human GRIP containing proteins outside the GRIP domain were identified outside metazoan taxa. In addition to the above methods, for these non-metazoan GRIP golgins, due to the short length and high sequence conservation of the GRIP domain, a bit score of 25 was used as a cutoff to identify positive hits, and criteria based on reverse search results were not applied. Results of the final searches, including accessions and E-values, are summarized in Additional file 6: Table S3. Alignments used for constructing HMMs are found in Additional file 9.

Finally, false negatives could be due to the divergence of a candidate from the experimentally validated *H. sapiens* query. In order to mitigate this possibility, HMMer searches were repeated with the same E-value cutoffs, but using protein databases of different taxa for reciprocal BLAST analysis. These taxa were selected from those taxa for which positive hits were validated in the previous HMMer searches, and which are included in the same supergroup as the taxa queried. For example, a CASP orthologue was identified in *Neospora caninum* using the closely related taxon *Toxoplasma gondii* for reverse BLAST searches, but not using *H. sapiens* (Additional file 6: Table S3). Additionally, BLAST was used to search nucleotide scaffold sequences in the case of one protein of interest (Sec16) in *Pichia pastoris* because it could not be found in the protein sequence database for this organism, and the protein database for the very closely related yeast *Komagataella phaffii* (which does contain a Sec16 sequence) was also included in the analyses.

### Phylogenetic analyses

For phylogenetic analyses, sequences were aligned using MUSCLE v3.8.31 [97] with default parameters, and manually trimmed to retain only regions of clear homology. Alignments used for phylogenetic analyses are found in Additional file 11 and Additional file 12. RAxML version 8.2.8 [98] was used for maximum likelihood analysis. For RAxML analyses, the PROTGAMMALG4X model was used, and 100 non-parametric bootstraps were performed using the default faster hill climbing method (–f b, –b, –N 100). MrBayes version 3.2.6 [99] was used for Bayesian analysis. For MrBayes analyses, over four million Markov chain Monte Carlo generations were run under the Mixed model with a burnin of 25% to average standard deviations of splits frequencies of 0.01 or lower, indicating convergence. Both RAxML and MrBayes analyses were run using the CIPRES webservice [100]. In the case of the GRASP proteins, several consecutive analyses were required with removal of divergent sequences to resolve phylogenetic relationships.

## Additional files

Additional file 1: **Table S1.** Human Golgi proteins examined, as well as their gene names, accession numbers, description of phenotype, and citations. (DOCX 147 kb)

Additional file 2: **Figure S1.** Dot plot of all potential Golgi stacking proteins examined. Taxa with unstacked Golgi are indicated by red text. Blue dots indicate identification of at least one orthologue. Light blue dots indicate the presence of an unresolved protein containing a GRIP-domain but which upon inspection of the alignment does not appear to be a confirmed orthologue of this protein. These proteins were therefore not taken into account when estimating the appearance point of a component. However, since all deductions made represent an estimate of “at least as early as time point X”, our deductions still stand, but origins of proteins could be slightly earlier than stated, should these candidates be real positive hits. Regardless, their presence does not affect the overall conclusions regarding pan-eukaryotic mechanisms of Golgi-stacking, since none of these cases involve ancient candidate stacking genes. For the GRIP-containing protein search results, positive hits in metazoans are also identified in searches specifically for the human GRIP domain-containing proteins GCC185, GCC88, golgin-245, or golgin-97. However, “GRIP-containing” includes animal-specific GRIP golgins (GCC88, GCC185, golgin-245, and golgin-97), as well as non-animal sequences with GRIP domains. Grey dots indicate identification of a potential GRIP domain-containing sequence not retrieved as positive hits in the previous searches, but matching the HMM with a bit score of at least 25. The striped dot (*P. pastoris* Sec16) indicates identification of Sec16 in nucleotide sequence scaffolds, but not predicted protein sequences (see Methods). Homology search results supporting the orthology assignments are shown in Additional file 6: Table S3. The phylogenetic tree on the left is based on established topologies for the taxa shown [75, 101]. (PDF 937 kb)

Additional file 3: **Table S2.** Annotated *M. balamuthi* genes encoding Golgi proteins. Predicted protein amino acid sequences of identified genes, after manual adjustment and annotation of gene models, are listed. BLAST search results are also listed for searches into *H. sapiens*, *S. cerevisiae*, and *D. discoideum* protein databases (Additional file 8: Figure S5) using the annotated *M. balamuthi* sequences as queries. (CSV 93 kb)

Additional file 4: **Figure S2.** Phylogenetic analysis of amoebozoan homologues of Adaptor protein complex and COPI complex β subunits used for classification of *M. balamuthi* genes within this paralogous family. Both MrBayes and RAxML were used in this analysis, yielding posterior probabilities and bootstrap values, respectively, as node support values, which are shown in the format MrBayes/RAxML (see Methods). The topology shown was reconstructed using MrBayes. Distinct clades for each of the proteins in this family were identified with significant support, allowing confident classification of *M. balamuthi* genes. The *M. balamuthi* sequences can be found in the alignment file used for this analysis (Additional file 11). (PDF 334 kb)

Additional file 5: **Figure S3.** Validation of antibodies used against *M. balamuthi*. Western blot analysis of *M. balamuthi* lysate and corresponding recombinant proteins using (A) anti-COPI-β and (B) anti-PDI Abs. (C) Immunofluorescence images of *M. balamuthi* incubated with pre-immune serum showing lack of fluorescence in the absence of the raised antibody. We speculate that, based on the estimated size of the larger band in panel A, the antibody is showing a dimer of the protein. In line with this, we performed preliminary proteomics of an SDS Page sample of proteins at the ~100 and ~200 KDa range. In both cases, we identified COPI-β as an abundant protein (data not shown). (PDF 14393 kb)

Additional file 6: **Table S3.** All potential Golgi stacking protein sequences identified. Some databases, including for *Homo sapiens* and *Rattus norvegicus*, include several predicted sequences for a single locus; therefore, each sequence does not necessarily correspond to a separate gene. (CSV 526 kb)

Additional file 7: **Figure S4.** Phylogenetic analysis of metazoan GRASP homologues indicates that the duplication producing the GRASP55 and GRASP65 paralogues occurred prior to the divergence of jawed fish from other vertebrates. Both MrBayes and RAxML were used in this analysis, yielding posterior probabilities and bootstrap values, respectively, as node support values, which are shown in the format MrBayes/RAxML (see Methods). The topology shown was reconstructed using MrBayes. Significant support was found for GRASP55 and GRASP65 clades, including *Callorhinchus milii* (Australian ghost shark) protein sequences, consistent with the presence of both paralogues in the ancestor of jawed fish and other vertebrates. GRASP protein sequences from earlier-branching metazoans do not split into distinct GRASP55 or GRASP65 clades, though they appear to share greater similarity with GRASP55 than GRASP65. (PDF 327 kb)

Additional file 8: **Figure S5.** Amino acid sequence alignments illustrating conservation of functional motifs of golgins (visualized using Boxshade). (A) C-terminal regions of selected GM130 and golgin-45 orthologues. (B) Segment of GRASP55 and GRASP65, and pre-duplicate GRASP alignment containing the position corresponding to Met164 of human GRASP65. (C) N-terminal region of identified GMAP210 orthologues showing loss of the N-terminal vesicle recognition motif in non-holozoan sequences, and loss of the ALPS domain in non-vertebrate sequences. (D) Conserved GRAB domain of GMAP210 orthologues from diverse eukaryotes including plants and metazoans. (E) Alignment of golgin-84 and CASP transmembrane domain sequences, which contain conserved residues. (F) N-terminal region of identified golgin-84 orthologues, showing comparable tryptophan-containing motifs in diverse eukaryotes. (G) Conserved Rab6-binding domain of TMF orthologues from eukaryotes including *Naegleria gruberi*. (PDF 212 kb)

Additional file 9: Amino acid sequence alignments used to construct Hidden Markov Models for homology searching. Alignment names correspond to HMM names in Additional file 6: Table S3. (AFA 5309 kb)

Additional file 10: **Table S4.** Sources of genomic data used for this study. (CSV 23 kb)

Additional file 11: Amino acid sequence alignment used for phylogenetic analysis of beta subunits of COPI and adaptin complexes (Additional file 4: Figure S2). The mask indicates the positions in the alignment that were included in the analysis. (AFA 40 kb)

Additional file 12: Amino acid sequence alignments used for phylogenetics analyses of GRASP homologues (Additional file 7: Figure S4). The mask indicates the positions in the alignment that were included in the analysis. (AFA 22 kb)
