# Correction to: A sophisticated, differentiated Golgi in the ancestor of eukaryotes

**DOI:** 10.1186/s12915-018-0510-y

**Published:** 2018-03-28

**Authors:** Lael D. Barlow, Eva Nývltová, Maria Aguilar, Jan Tachezy, Joel B. Dacks

**Affiliations:** 1 Department of Cell Biology, Faculty of Medicine and Dentistry, University of Alberta, 5-31 Medical Sciences Building, Edmonton, Alberta T6G 2H7 Canada; 2 Department of Parasitology (BIOCEV), Faculty of Science, Charles University, Průmyslová 595, 252 42 Vestec, Czech Republic; 3 Department of Neurology, University of Miami Miller School of Medicine, 1600 NW 10th Avenue, Rosenstiel Medical Science Building (RMSB) # 2067, Miami, Florida 33136 USA; 4 Department of Life Sciences, The Natural History Museum, Cromwell Road, London, SW7 5BD UK

## Correction

Upon publication of the original article, Barlow et al. [1], the authors noticed that Fig. 4b contained an inaccuracy when additional data is taken into account. We inferred a loss of GRASP in the common ancestor of cryptophytes and archaeplastids, based on the absence of identified homologues in the data from taxa that we analyzed, which include *Cyanidioschyzon merolae* as the single representative of red algae. However, this inference is incorrect when additional red algal taxa are considered. Using the same methods as in the original paper, we identified the following sequences in sequence data from other red algae: *Chondrus crispus* (XP_005713669.1), *Galdieria sulphuraria* (XP_005704721.1 and XP_005704722.1), *Porphyra umbilicalis* (OSX69770.1), and *Porphyridium purpureum* (evm.model.contig_2019.4 from http://cyanophora.rutgers.edu/porphyridium/). Therefore, the ancestor of Archaeplastida plus Cryptophyta likely possessed a GRASP homologue, and multiple losses likely occurred, including in cryptophytes, glaucophytes, and *Cyanidioschyzon*. However, because cryptophytes and glaucophytes are represented in the analysis only by one exemplar genome per lineage, loss of a GRASP gene cannot be strongly inferred (and is thus not shown in Fig. 4b). Importantly, these additional results are still consistent with the published conclusions that the last eukaryotic common ancestor possessed a GRASP homologue, and that the presence of such homologues across eukaryotic diversity does not correlate with stacked Golgi morphology.

**Fig. 4:**
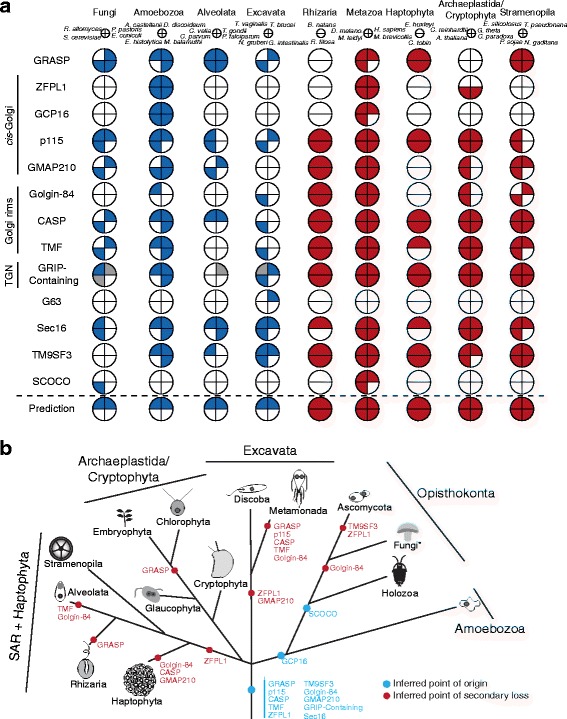
Pan-eukaryotic Golgi protein evolution. **a** Coulson plot of Golgi proteins found outside the Metazoa. Most importantly, while these represent ancient proteins, none show the phylogenetic pattern that would be expected for a necessary stacking factor, illustrated in the “Prediction” row. To clarify the patterns of presence and absence in organisms with stacked and unstacked cisternae, only selected genomes are shown here. The full data are given in Additional file 2: Figure S1 and Additional file 6: Table S3. The first four columns (blue) show genes identified in organisms with unstacked Golgi, and closely related organisms with stacked Golgi, while remaining columns (red) indicate genes identified in representatives of taxonomic groups with stacked Golgi. Gray sectors indicate sequences identified using alternative methods (Additional file 2: Figure S1). **b** Schematic showing the timing of gains and losses of the proteins across eukaryotic evolution. Note that, if a single member of the taxonomic group possesses an orthologue of the protein, it is inferred as present in that group. Relationships between eukaryotes are based on recent concatenated phylogenetic results [75, 101]. To highlight losses in the Ascomycota, they are broken out to the exclusion of the paraphyletic remaining Fungi (denoted by the asterisk)

Please see the corrected figure below (Fig. 4b).

Furthermore, while the last sentence of the second paragraph in the “Evolution of the interacting Golgi structural proteins GM130, golgin-45, GRASP55, and GRASP65” subsection of the Results section in the original manuscript reads

“However, GRASP was not identified in many cases, most prominently in Embryophyta as previously noted [33] and extended here to the entire clade of Archaeplastida plus Cryptophyta, as well as Rhizaria and Metamonada (Fig. 4).”,

it should in fact be

“However, GRASP was not identified in many cases, most prominently in Embryophyta as previously noted [33], and independently in Cryptophyta (*Guillardia theta*) as well as Rhizaria and Metamonada (Fig. 4).”
