# Supplementary material for: A sophisticated, differentiated Golgi in the ancestor of eukaryotes
Source: BMC Biol. 2018 Mar 7;16:27. doi: 10.1186/s12915-018-0492-9 (PMC5840792; doi:10.1186/s12915-018-0492-9)
Supplement: Supplementary file 1 — Table S1. Human Golgi proteins examined, as well as their gene names, accession numbers, description of phenotype, and citations. (DOCX 147 kb) [file 12915_2018_492_MOESM1_ESM.docx]

**Barlow et al. Table S1**

| **Protein name** | **Protein accession** | **Gene name** | **Description** | **References** |
| --- | --- | --- | --- | --- |
| Acyl-coenzyme A binding domain containing 3 protein (ACBD3) (a.k.a. Golgi complex-associated protein of 60 kDa (GCP60)) | NP_073572.2 | ACBD3 | Interacts with Giantin. Overexpression resulted in Golgi disassembly. Has numerous binding partners, and is implicated in numerous cellular functions. | [1, 2] |
| Centrosome and Golgi localized PKN-associated protein (CG-NAP) (a.k.a. A-kinase anchoring protein 9) | NP_005742.4 | AKAP9 | Localizes to centrosome and Golgi, and plays a role in microtubule nucleation. Anchored to Golgi via interaction with GM130. | [3–5] |
| Bicaudal-D1, Bicaudal-D2 | NP_001705.2, NP_001003800.1 | BICD1, BICD2 | Bicaudal-D2 interacts with Dynein. | [6, 7] |
| Cytohesin-associated scaffolding protein (CASP) | NP_853530.2 | CUX1 | Localized to Golgi rims and COPI vesicles, anchored by a transmembrane domain. Binds to Golgin-84. | [8] |
| Giantin | NP_001243415.1 | GOLGB1 | Localized to Golgi rims. Involved in organizing Golgi stacks. Interacts with p115. | [9–12] |
| Golgi complex associated protein of 16 kD (GCP16) | NP_057183.2 | GOLGA7 | Interacts with Golgin-160 at the Golgi membrane. | [13] |
| Golgi localized coiled-coil protein of 185 kD (GCC185) | NP_852118.1 | GCC2 | Binds Arl1 and Rab6 at *trans*-Golgi/TGN. Involved in maintaining Golgi structure, and organizing microtubules. | [14–17] |
| Golgi localized coiled-coil protein of 88 kD (GCC88) | NP_078799.2 | GCC1 | Binds Arl1 at *trans*-Golgi/TGN. Involved in vesicle trafficking from endosomes to Golgi. | [16, 18] |
| Golgi matrix protein of 130 kD (GM130) | NP_004477.3 | GOLGA2 | *Cis*-Golgi localization. Contributes to Golgi stacking. Involved in vesicle trafficking from ER to Golgi. Binds to GRASP65. Also connects the Golgi to microtubules. | [18–22] |
| Golgi microtubule-associated protein 210 (GMAP210) | NP_004230.2 | TRIP11 | *Cis*-Golgi localization. Tethers vesicles from the ER, and the medial/early Golgi. | [18, 23] |
| Golgi Reassembly and Stacking Protein of 55 kD (GRASP55) | NP_001188357.1 | GORASP2 | Localized to *medial*-Golgi cisternae. Stacks/unstacks Golgi cisternae in mammalian cells. | [19, 24] |
| Golgi Reassembly and Stacking Protein of 65 kD (GRASP65) | NP_114105.1 | GORASP1 | Localized to *cis*-Golgi cisternae. Stacks/unstacks Golgi cisternae in mammalian cells. | [19, 25] |
| Golgin-160 | NP_005886.2 | GOLGA3 | Cis-Golgi localization. Recruits dynein for positioning of Golgi cisternae along microtubules. | [26, 27] |
| Golgin-245 | NP_001166184.1 | GOLGA4 | Binds Arl1 at *trans*-Golgi/TGN. Involved in vesicle trafficking from endosomes to Golgi. | [18] |
| Golgin-45 | NP_001307902.1 | BLZF1 | Contributes to Golgi stacking in Human cells. Binds to GRASP55. | [19] |
| Golgin-67 | NP_851422.1 | GOLGA8A | Unknown function at the Golgi. | [28, 29] |
| Golgin-84 | NP_005104.3 | GOLGA5 | Localized to Golgi rims, anchored by a transmembrane domain. Binds to CASP and Rab1, and is involved in Golgi ribbon formation (lateral connection of stacks) and stacking. Tethers vesicles from the late/medial Golgi. | [8, 18, 30–32] |
| Golgin-97 | NP_002068.1 | GOLGA1 | Binds Arl1 at *trans*-Golgi/TGN. Involved in vesicle trafficking from endosomes to Golgi. | [18] |
| Neuroendocrine long coiled-coil protein 1 and 2 (NECC1, NECC2) | NP_055605.2, NP_001098991.1 | JAKMIP2, JAKMIP3 | Primarily expressed in neuroendocrine tissues of vertebrates, and related to a third paralogue, JAKMIP1. Knockdown does not affect Golgi structure. NECC1 modulates secretion. | [33, 34] |
| p115 | NP_001276978.1 | USO1 | Localized to the *cis*-Golgi. Proposed to be involved in stacking cisternae, vesicle tethering, interaction with gamma tubulin, and interaction with Giantin, GM130, and Golgi SNAREs. | [35–39] |
| SCY1-like 1 binding protein 1 (SCYL1BP1) | NP_689494.2 | GORAB | Localizes to the *trans*-Golgi/TGN, contains coiled-coil regions, and is regulated by Rab6, but has poorly understood function. | [40] |
| Sec16A, Sec16B | NP_055681.1, NP_149118.2 | SEC16A, SEC16B | Involved in ER export through interaction with other COPII complex components, and a homologue is involved in Golgi stacking in *Pichia pastoris*. | [41, 42] |
| Short coiled-coil (SCOCO) | NP_001146956.1 | SCOC | Binds to Arl1 (likely at the *trans*-Golgi/TGN) in mammalian cells. Has a previously identified homologue (Slo1p) in yeast. Unknown function. | [43, 44] |
| TATA element modulatory factor (TMF) | NP_009045.2 | TMF1 | Localized to Golgi rims near the *trans* side. Involved in vesicle trafficking between Golgi cisternae. Knockdown results in dispersion of Golgi membranes. | [18, 45–47] |
| Transmembrane 9 superfamily member 3 (TM9SF3) | NP_064508.3 | TM9SF3 | Present on Golgi (stacked) but not acrosome (unstacked Golgi-derived compartment) of developing spermatids. | [48] |
| Zinc finger protein-like 1 (ZFPL1) | NP_006773.2 | ZFPL1 | Binds to GM130. Depletion disrupts *cis*-Golgi structure. | [49] |

**References for Table S1:**

1. Sohda M, Misumi Y, Yamamoto a, Yano a, Nakamura N, Ikehara Y. Identification and characterization of a novel Golgi protein, GCP60, that interacts with the integral membrane protein giantin. J Biol Chem. 2001;276:45298–306. doi:10.1074/jbc.M108961200.

2. Fan J, Liu J, Culty M, Papadopoulos V. Acyl-coenzyme A binding domain containing 3 (ACBD3; PAP7; GCP60): An emerging signaling molecule. Prog Lipid Res. 2010;49:218–34. doi:10.1016/j.plipres.2009.12.003.

3. Takahashi M, Shibata H, Shimakawa M, Miyamoto M, Mukai H, Yoshitaka O. Characterization of a novel giant scaffolding protein, CG-NAP, that anchors multiple signaling enzymes to centrosome and the Golgi apparatus. J Biol Chem. 1999;274:17267–74.

4. Rios RM. The centrosome – Golgi apparatus nexus. Phil Trans R Soc B. 2014;369.

5. Rivero S, Cardenas J, Bornens M, Rios RM. Microtubule nucleation at the cis-side of the Golgi apparatus requires AKAP450 and GM130. EMBO J. 2009;28:1016–28.

6. Hoogenraad CC, Akhmanova A, Howell SA, Dortland BR, Zeeuw CI De, Willemsen R, et al. Mammalian Golgi-associated Bicaudal-D2 functions in the dynein ± dynactin pathway by interacting with these complexes. EMBO J. 2001;20:4041–54.

7. Hoogenraad CC, Wulf P, Schiefermeier N, Stepanova T, Galjart N, Small JV, et al. Bicaudal D induces selective dynein-mediated microtubule minus end-directed transport. EMBO J. 2003;22:6004–15.

8. Malsam J, Satoh A, Pelletier L, Warren G. Golgin tethers define subpopulations of COPI vesicles. Science. 2005;307:1095–8.

9. Koreishi M, Gniadek TJ, Yu S, Masuda J, Honjo Y, Satoh A. The golgin tether giantin regulates the secretory pathway by controlling stack organization within Golgi apparatus. PLoS One. 2013;8:e59821. doi:10.1371/journal.pone.0059821.

10. Petrosyan A, Holzapfel MS, Muirhead DE, Cheng P-W. Restoration of compact golgi morphology in advanced prostate cancer enhances susceptibility to galectin-1-induced apoptosis by modifying mucin O-glycan synthesis. Mol Cancer Res. 2014;12:1704–16. doi:10.1158/1541-7786.MCR-14-0291-T.

11. Linstedt AD, Hauri HP. Giantin, a novel conserved Golgi membrane protein containing a cytoplasmic domain of at least 350 kDa. Mol Biol Cell. 1993;4:679–93.

12. Lesa GM, Seemann J, Shortert J, Vandekerckhove J, Warren G. The amino-terminal domain of the golgi protein Giantin interacts directly with the vesicle-tethering protein p115. J Biol Chem. 2000;275:2831–6.

13. Ohta E, Misumi Y, Sohda M, Fujiwara T, Yano A, Ikehara Y. Identification and Characterization of GCP16, A Novel Acylated Golgi Protein That Interacts with GCP170. J Biol Chem. 2003;278:51957–67.

14. Efimov A, Kharitonov A, Efimova N, Loncarek J, Miller PM, Andreyeva N, et al. Asymmetric CLASP-Dependent Nucleation of Noncentrosomal Microtubules at the trans-Golgi Network. Dev Cell. 2007;12:917–30.

15. Derby MC, Lieu ZZ, Brown D, Stow JL, Goud B, Gleeson PA. The trans-Golgi network golgin, GCC185, is required for endosome-to-golgi transport and maintenance of Golgi structure. Traffic. 2007;8:758–73.

16. Luke MR, Kjer-Nielsen L, Brown DL, Stow JL, Gleeson PA. GRIP domain-mediated targeting of two new coiled-coil proteins, GCC88 and GCC185, to subcompartments of the trans-Golgi network. J Biol Chem. 2003;278:4216–26.

17. Burguete AS, Fenn TD, Brunger AT, Pfeffer SR. Rab and Arl GTPase Family Members Cooperate in the Localization of the Golgin GCC185. Cell. 2008;132:286–98.

18. Wong M, Munro S. The specificity of vesicle traffic to the Golgi is encoded in the golgin coiled-coil proteins. Science (80- ). 2014.

19. Lee I, Tiwari N, Dunlop MH, Graham M, Liu X, Rothman JE. Membrane adhesion dictates Golgi stacking and cisternal morphology. Proc Natl Acad Sci U S A. 2014;111:1849–54. doi:10.1073/pnas.1323895111.

20. Cohen-kupiec R, Zilberstein A, Gurevitz M. Characterization of cis-Golgi Matrix Protein, GM130. Microbiology. 1995;177:2222–6.

21. Kodani A, Sutterlin C. The Golgi Protein GM130 Regulates Centrosome Morphology and Function. Mol Biol Cell. 2008;19 February:745–53.

22. Rivero S, Cardenas J, Bornens M, Rios RM. Microtubule nucleation at the cis-side of the Golgi apparatus requires AKAP450 and GM130. EMBO J. 2009;28:1016–28. doi:10.1038/emboj.2009.47.

23. Infante C, Ramos-morales F, Fedriani C, Bornens M, Rios RM. GMAP-210, A Cis-Golgi Network-associated Protein, Is a Minus End Microtubule-binding Protein. J Cell Biol. 1999;145:83–98.

24. Shorter J, Watson R, Giannakou ME, Clarke M, Warren G, Barr FA. GRASP55, a second mammalian GRASP protein involved in the stacking of Golgi cisternae in a cell-free system. EMBO J. 1999;18:4949–60.

25. Barr FA, Puype M, Warren G. GRASP65 , a Protein Involved in the Stacking of Golgi Cisternae. Cell. 1997;91:253–62.

26. Fritzler MJ, Hamel JC, Ochs RL, Chan EKL. Molecular Characterization of Two Human Autoantigens: Unique cDNAs Encoding 95- and 160-kD Proteins of a Putative Family in the Golgi Complex. J Exp Med. 1993;178 July:49–62.

27. Yadav S, Puthenveedu MA, Linstedt AD. Golgin160 Recruits the Dynein Motor to Position the Golgi Apparatus. Dev Cell. 2012;23:153–65.

28. Jakymiw A, Raharjo E, Rattner JB, Eystathioy T, Chan EKL, Fujita DJ. Identification and characterization of a novel Golgi protein, golgin-67. J Biol Chem. 2000;275:4137–44.

29. Eystathioy T, Jakymiw A, Fujita DJ, Fritzler MJ, Chan EK. Human autoantibodies to a novel Golgi protein golgin-67: high similarity with golgin-95/gm 130 autoantigen. J Autoimmun. 2000;14:179–87. doi:10.1006/jaut.1999.0359.

30. Diao A, Rahman D, Pappin DJC, Lucocq J, Lowe M. The coiled-coil membrane protein golgin-84 is a novel rab effector required for Golgi ribbon formation. J Cell Biol. 2003;160:201–12. doi:10.1083/jcb.200207045.

31. Sohda M, Misumi Y, Yamamoto A, Nakamura N, Ogata S, Sakisaka S, et al. Interaction of Golgin-84 with the COG Complex Mediates the Intra-Golgi Retrograde Transport. Traffic. 2010;11:1552–66.

32. Satoh A, Wang Y, Malsam J, Beard MB, Warren G. Golgin-84 is a rab1 binding partner involved in Golgi structure. Traffic. 2003;4:153–61. doi:10.1034/j.1600-0854.2003.00103.x.

33. Cruz-Garcia D, Vazquez-Martinez R, Peinado JR, Anouar Y, Tonon MC, Vaudry H, et al. Identification and characterization of two novel (neuro)endocrine long coiled-coil proteins. FEBS Lett. 2007;581:3149–56.

34. Cruz‑García D, Díaz‑Ruiz A, Rabanal‑Ruiz Y, Peinado JR, Gracia‑Navarro F, Castaño JP, et al. The Golgi-associated long coiled-coil protein NECC1 participates in the control of the regulated secretory pathway in PC12 cells. Biochem J. 2012;443:387–96.

35. Shorter J, Warren G. A role for the vesicle tethering protein, p115, in the post-mitotic stacking of reassembling Golgi cisternae in a cell-free system. J Cell Biol. 1999;146:57–70. http://www.pubmedcentral.nih.gov/articlerender.fcgi?artid=2199741&tool=pmcentrez&rendertype=abstract.

36. Shorter J, Beard MB, Seemann J, Barbara Dirac-Svejstrup A, Warren G. Sequential tethering of Golgins and catalysis of SNAREpin assembly by the vesicle-tethering protein p115. J Cell Biol. 2002;157:45–62.

37. Puthenveedu MA, Linstedt AD. Evidence that Golgi structure depends on a p115 activity that is independent of the vesicle tether components giantin and GM130. J Cell Biol. 2001;155:227–38. doi:10.1083/jcb.200105005.

38. Radulescu AE, Mukherjee S, Shields D. The golgi protein p115 associates with γ-tubulin and plays a role in golgi structure and mitosis progression. J Biol Chem. 2011;286:21915–26.

39. Linstedt AD, Jesch SA, Mehta A, Lee TH, Garcia-mata R, Nelson DS, et al. Binding Relationships of Membrane Tethering Components. J Biol Chem. 2000;275:10196–201.

40. Hennies HC, Kornak U, Zhang H, Egerer J, Zhang X, Seifert W, et al. Gerodermia osteodysplastica is caused by mutations in SCYL1BP1, a Rab-6 interacting golgin. Nat Genet. 2008;40:1410–2. doi:10.1038/ng.252.

41. Connerly PL, Esaki M, Montegna E a, Strongin DE, Levi S, Soderholm J, et al. Sec16 is a determinant of transitional ER organization. Curr Biol. 2005;15:1439–47. doi:10.1016/j.cub.2005.06.065.

42. Bharucha N, Liu Y, Papanikou E, McMahon C, Esaki M, Jeffrey PD, et al. Sec16 influences transitional ER sites by regulating rather than organizing COPII. Mol Biol Cell. 2013;24:3406–19. doi:10.1091/mbc.E13-04-0185.

43. Van Valkenburgh H, Shern JF, Sharer JD, Zhu X, Kahn RA. ADP-ribosylation factors (ARFs) and ARF-like 1 (ARL1) have both specific and shared effectors. Characterizing ARL1-binding proteins. J Biol Chem. 2001;276:22826–37.

44. Panic B, Whyte JRC, Munro S. The ARF-like GTPases Arl1p and Arl3p act in a pathway that interacts with vesicle-tethering factors at the Golgi apparatus. Curr Biol. 2003;13:405–10.

45. Mori K, Kato H. A putative nuclear receptor coactivator (TMF/ARA160) associates with hbrm/hSNF2α and BRG-1/hSNF2β and localizes in the Golgi apparatus. FEBS Lett. 2002;520:127–32.

46. Fridmann-Sirkis Y, Siniossoglou S, Pelham HRB. TMF is a golgin that binds Rab6 and influences Golgi morphology. BMC Cell Biol. 2004;5:18. doi:10.1186/1471-2121-5-18.

47. Yamane J, Kubo A, Nakayama K, Yuba-Kubo A, Katsuno T, Tsukita S, et al. Functional involvement of TMF/ARA160 in Rab6-dependent retrograde membrane traffic. Exp Cell Res. 2007;313:3472–85.

48. Au CE, Hermo L, Byrne E, Smirle J, Fazel A, Simon PHG, et al. Expression, sorting, and segregation of Golgi proteins during germ cell differentiation in the testis. Mol Biol Cell. 2015;26:4015–32. doi:10.1091/mbc.E14-12-1632.

49. Chiu C-F, Ghanekar Y, Frost L, Diao A, Morrison D, McKenzie E, et al. ZFPL1, a novel ring finger protein required for cis-Golgi integrity and efficient ER-to-Golgi transport. EMBO J. 2008;27:934–47. doi:10.1038/emboj.2008.40.
