# Supplementary figures and images for: A sophisticated, differentiated Golgi in the ancestor of eukaryotes
Source: BMC Biol. 2018 Mar 7;16:27. doi: 10.1186/s12915-018-0492-9 (PMC5840792; doi:10.1186/s12915-018-0492-9)

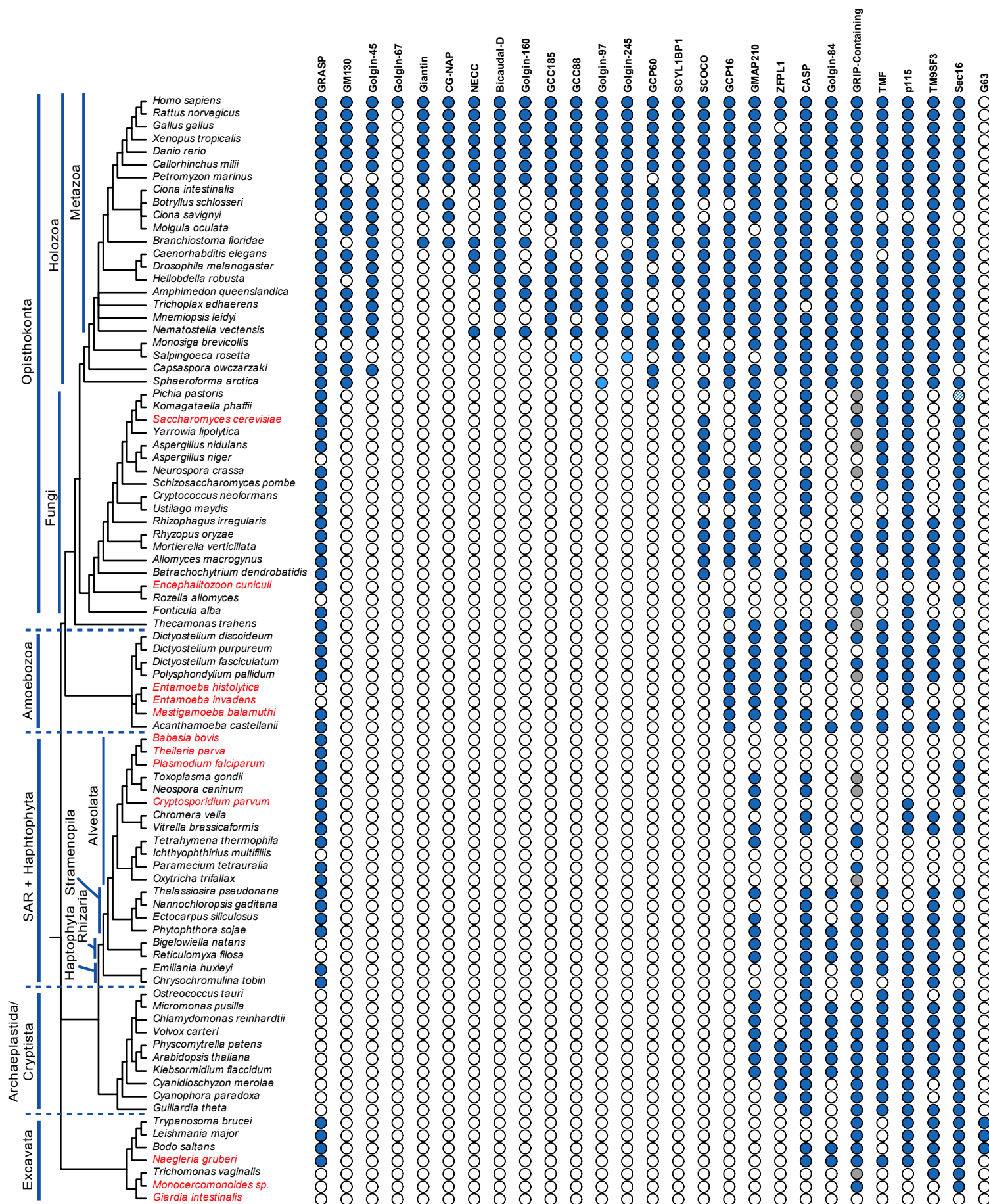

Barlow et al. Figure S1

Supplement: Supplementary file 2 — Figure S1. Dot plot of all potential Golgi stacking proteins examined. Taxa with unstacked Golgi are indicated by red text. Blue dots indicate identification of at least one orthologue. Light blue dots indicate the presence of an unresolved protein containing a GRIP-domain but which upon inspection of the alignment does not appear to be a confirmed orthologue of this protein. These proteins were therefore not taken into account when estimating the appearance point of a component. However, since all deductions made represent an estimate of “at least as early as time point X”, our deductions still stand, but origins of proteins could be slightly earlier than stated, should these candidates be real positive hits. Regardless, their presence does not affect the overall conclusions regarding pan-eukaryotic mechanisms of Golgi-stacking, since none of these cases involve ancient candidate stacking genes. For the GRIP-containing protein search results, positive hits in metazoans are also identified in searches specifically for the human GRIP domain-containing proteins GCC185, GCC88, golgin-245, or golgin-97. However, “GRIP-containing” includes animal-specific GRIP golgins (GCC88, GCC185, golgin-245, and golgin-97), as well as non-animal sequences with GRIP domains. Grey dots indicate identification of a potential GRIP domain-containing sequence not retrieved as positive hits in the previous searches, but matching the HMM with a bit score of at least 25. The striped dot (P. pastoris Sec16) indicates identification of Sec16 in nucleotide sequence scaffolds, but not predicted protein sequences (see Methods). Homology search results supporting the orthology assignments are shown in Additional file 6: Table S3. The phylogenetic tree on the left is based on established topologies for the taxa shown [75, 101]. (PDF 937 kb) [file 12915_2018_492_MOESM2_ESM.pdf]

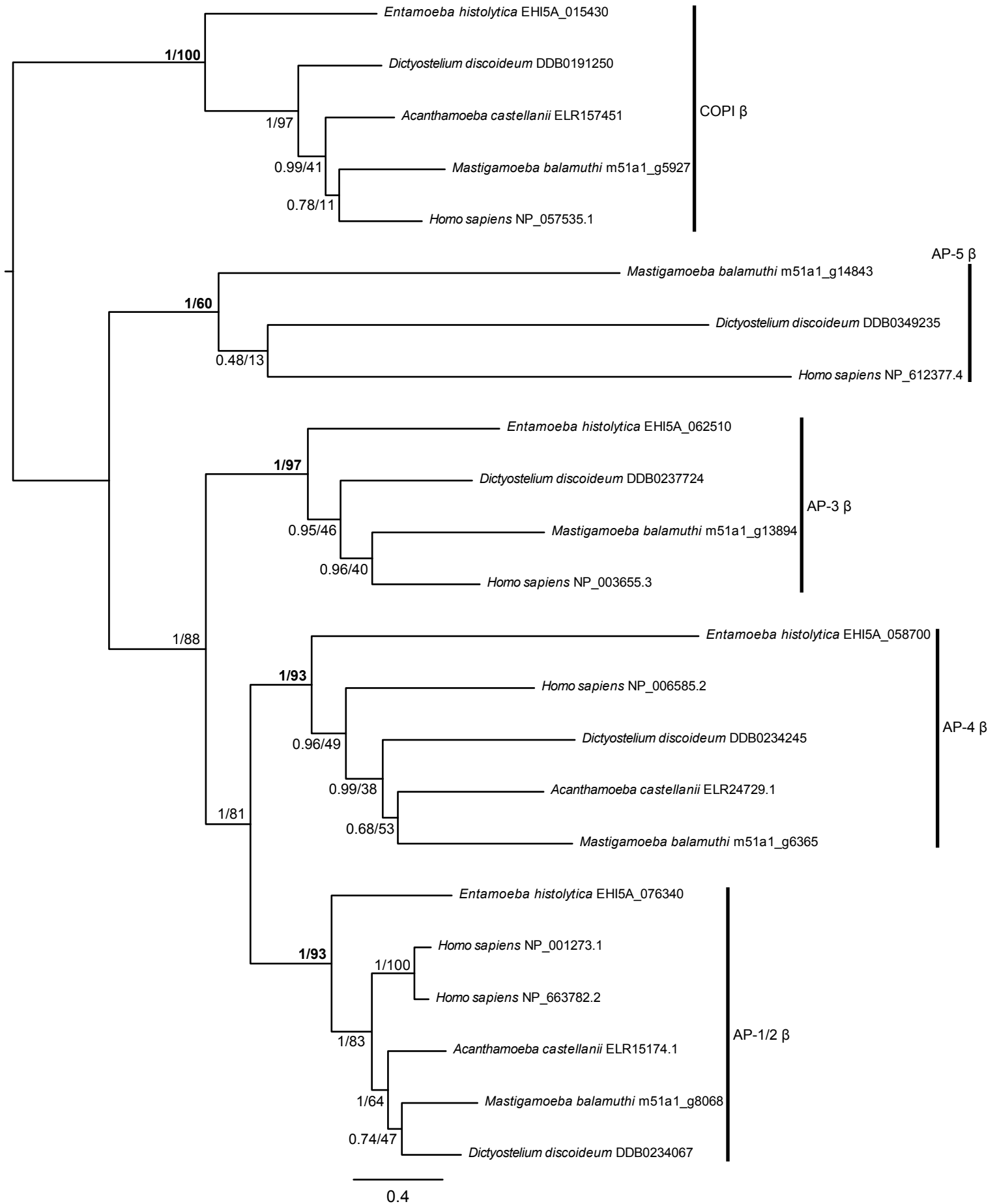

Supplement: Supplementary file 4 — Figure S2. Phylogenetic analysis of amoebozoan homologues of Adaptor protein complex and COPI complex β subunits used for classification of M. balamuthi genes within this paralogous family. Both MrBayes and RAxML were used in this analysis, yielding posterior probabilities and bootstrap values, respectively, as node support values, which are shown in the format MrBayes/RAxML (see Methods). The topology shown was reconstructed using MrBayes. Distinct clades for each of the proteins in this family were identified with significant support, allowing confident classification of M. balamuthi genes. The M. balamuthi sequences can be found in the alignment file used for this analysis (Additional file 11). (PDF 334 kb) [file 12915_2018_492_MOESM4_ESM.pdf]

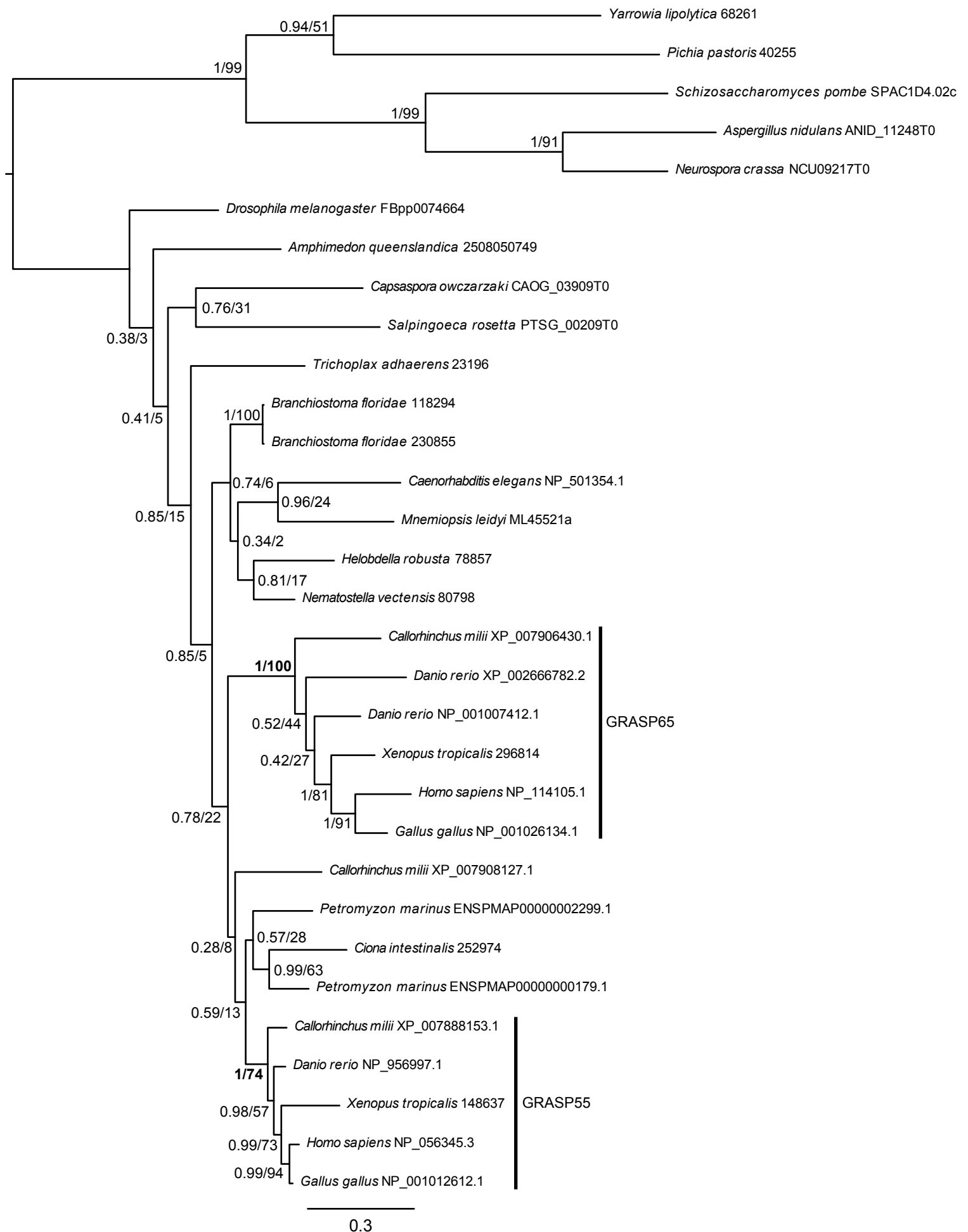

Supplement: Supplementary file 7 — Figure S4. Phylogenetic analysis of metazoan GRASP homologues indicates that the duplication producing the GRASP55 and GRASP65 paralogues occurred prior to the divergence of jawed fish from other vertebrates. Both MrBayes and RAxML were used in this analysis, yielding posterior probabilities and bootstrap values, respectively, as node support values, which are shown in the format MrBayes/RAxML (see Methods). The topology shown was reconstructed using MrBayes. Significant support was found for GRASP55 and GRASP65 clades, including Callorhinchus milii (Australian ghost shark) protein sequences, consistent with the presence of both paralogues in the ancestor of jawed fish and other vertebrates. GRASP protein sequences from earlier-branching metazoans do not split into distinct GRASP55 or GRASP65 clades, though they appear to share greater similarity with GRASP55 than GRASP65. (PDF 327 kb) [file 12915_2018_492_MOESM7_ESM.pdf]
