# Supplementary material for: A sophisticated, differentiated Golgi in the ancestor of eukaryotes
Source: BMC Biol. 2018 Mar 7;16:27. doi: 10.1186/s12915-018-0492-9 (PMC5840792; doi:10.1186/s12915-018-0492-9)
Supplement: Supplementary file 5 — Figure S3. Validation of antibodies used against M. balamuthi. Western blot analysis of M. balamuthi lysate and corresponding recombinant proteins using (A) anti-COPI-β and (B) anti-PDI Abs. (C) Immunofluorescence images of M. balamuthi incubated with pre-immune serum showing lack of fluorescence in the absence of the raised antibody. We speculate that, based on the estimated size of the larger band in panel A, the antibody is showing a dimer of the protein. In line with this, we performed preliminary proteomics of an SDS Page sample of proteins at the ~100 and ~200 KDa range. In both cases, we identified COPI-β as an abundant protein (data not shown). (PDF 14393 kb) [file 12915_2018_492_MOESM5_ESM.pdf]

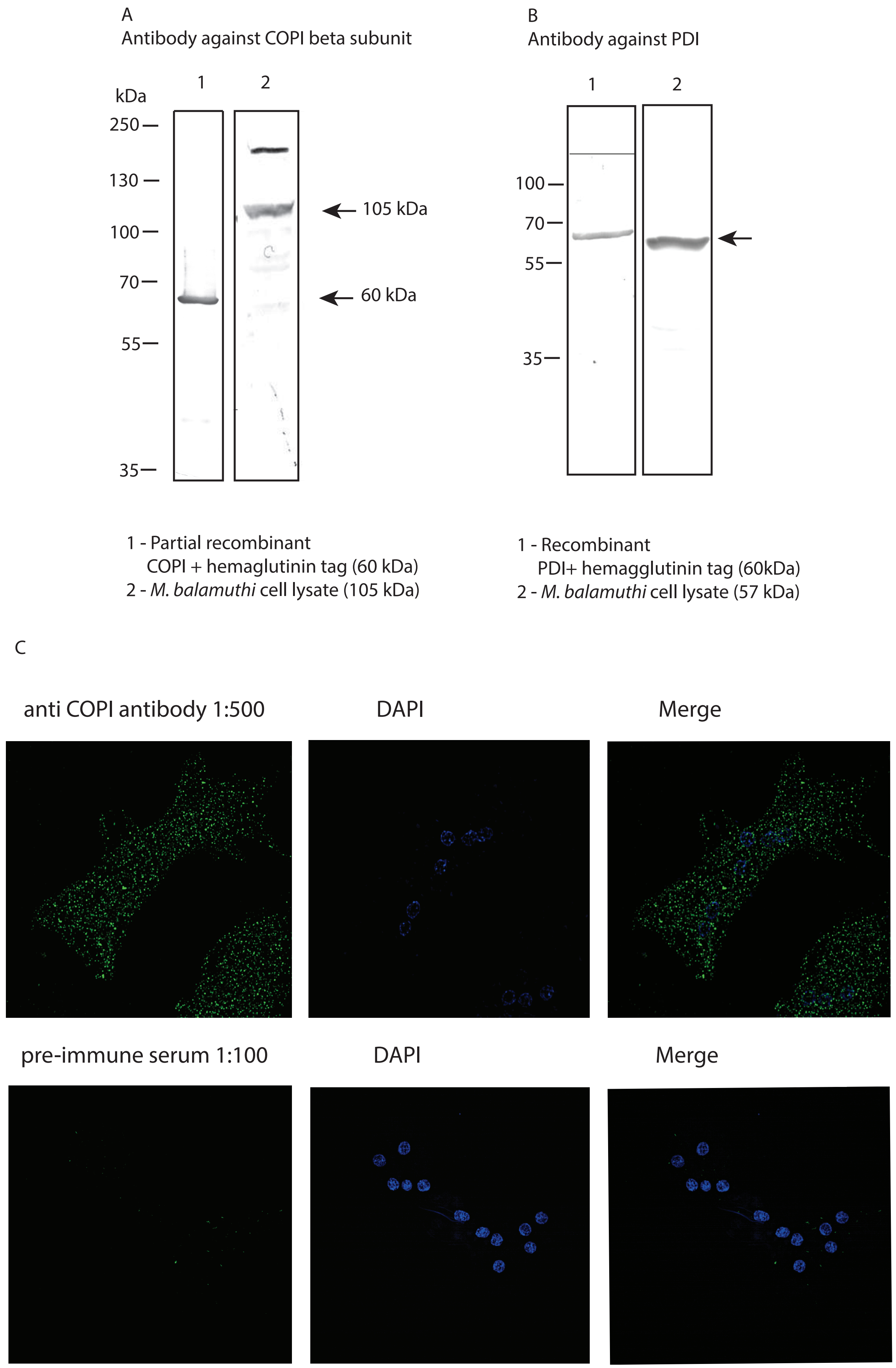

Barlow et al. Figure S3
