# Supplementary material for: A sophisticated, differentiated Golgi in the ancestor of eukaryotes
Source: BMC Biol. 2018 Mar 7;16:27. doi: 10.1186/s12915-018-0492-9 (PMC5840792; doi:10.1186/s12915-018-0492-9)
Supplement: Supplementary file 8 — Figure S5. Amino acid sequence alignments illustrating conservation of functional motifs of golgins (visualized using Boxshade). (A) C-terminal regions of selected GM130 and golgin-45 orthologues. (B) Segment of GRASP55 and GRASP65, and pre-duplicate GRASP alignment containing the position corresponding to Met164 of human GRASP65. (C) N-terminal region of identified GMAP210 orthologues showing loss of the N-terminal vesicle recognition motif in non-holozoan sequences, and loss of the ALPS domain in non-vertebrate sequences. (D) Conserved GRAB domain of GMAP210 orthologues from diverse eukaryotes including plants and metazoans. (E) Alignment of golgin-84 and CASP transmembrane domain sequences, which contain conserved residues. (F) N-terminal region of identified golgin-84 orthologues, showing comparable tryptophan-containing motifs in diverse eukaryotes. (G) Conserved Rab6-binding domain of TMF orthologues from eukaryotes including Naegleria gruberi. (PDF 212 kb) [file 12915_2018_492_MOESM8_ESM.pdf]

```
                                                                          F975           I990
                                                                          *              *
Homo sapiens GM130 AAF65550.1                  958   MQNPRERPG--L---GSNPCIPFFYRADENDEVKITVI
Gallus gallus GM130 XP_004945878.1            1017   IQNPQERSGSLL----ENPCIPFFYRADENDEVKIMVV
Xenopus tropicalis GM130 453473                922   IQNPQERPSALL----QNPCIPFFYRADENDEVRIMVI
Danio rerio GM130 XP_009299396.1              1022   IQNPQSRPAPFL---GENPCIPFFYRPDEHDEVKILVV
Callorhinchus milii GM130 XP_007898513.1       997   IQNPQAKQSPFL---SYSPCIPFFYRADEHDEVRILVV
Ciona intestinalis GM130 291865                581   MQNPSFMNSDKA---PRGFCLRYM---------GRYTAV
Drosophila melanogaster GM130 FBpp0071703      769   IKENTEQPSHNHAGDHLNCCM-----------GKFEVV
Caenorhabditis elegans GM130 NP_494929.3       905   ISRPQILPAGQL---HCTQCI-----------GDLQEL
Amphimedon queenslandica GM130 2508048534      563   LTSPLTSIEPNV---HCPCCS-----------GNLIIV
Mnemiopsis leidyi GM130 ML05262a               686   TDVERKQVSGPM---YAGGR------------GPIKDF
Salpingoeca rosetta GM130 PTSG_06277T0         404   QQRQRARVPEWV---QAATSHGYYAK------FSVMSL
Capsaspora owczarzaki GM130 CAOG_04888T0       930   MLDSIEGTATSIEFLPCKKCS-----------ATVQVV
Sphaeroforma arctica GM130 SARC_07317T0        893   -KDTQKVPIGIQ---HVVPI------------------
```

Barlow et al. Figure S5A

		Met164 *
Homo sapiens GRASP65 NP_114105.1	160	PLKLMVYN
Xenopus tropicalis GRASP65 296814	162	PLKLLVYN
Gallus gallus GRASP65 NP_001026134.1	160	PLKLMVYN
Danio rerio GRASP65 XP_002666782.2	157	SLKLLVYN
Danio rerio GRASP65 NP_001007412.1	161	PLKLLIYN
Callorhinchus milii GRASP65 XP_007906430.1	161	PLKLLVYN
Homo sapiens GRASP55 NP_056345.3	160	PLKLYVYN
Xenopus tropicalis GRASP55 148637	160	PLKLYVYN
Gallus gallus GRASP55 NP_001012612.1	160	PLKLYVYN
Danio rerio GRASP55 NP_956997.1	160	GLKLYVYN
Callorhinchus milii GRASP55 XP_007888153.1	160	PLKLYVYN
Callorhinchus milii GRASP XP_007908127.1	181	PLKIYVYN
Petromyzon marinus GRASP ENSPMAP00000002299.1	112	PLKLYVYN
Petromyzon marinus GRASP ENSPMAP00000000179.1	160	PLKLYVYN
Ciona intestinalis GRASP 252974	160	QLKLFVYN
Branchiostoma floridae GRASP 118294	160	PLKLYVYN
Branchiostoma floridae GRASP 230855	160	PLKLYVYN
Caenorhabditis elegans GRASP NP_501354.1	160	PLKLYVYN
Drosophila melanogaster GRASP FBpp0074664	160	PLKMYVYN
Hellobdella robusta GRASP 78857	160	PLKLYVYN
Amphimedon queenslandica GRASP 2508050749	160	TIRLFVYN
Nematostella vectensis GRASP 80798	160	PLKLYVYN
Trichoplax adhaerens GRASP 23196	161	PIRLYVYS
Mnemiopsis leidyi GRASP ML45521a	159	PIKVYVYS
Capsaspora owczarzaki GRASP CAOG_03909T0	164	ALSLFVYN
Salpingoeca rosetta GRASP PTSG_00209T0	159	PLRLYVYN
Aspergillus nidulans GRASP ANID_11248T0	167	TLVLWVYN
Neurospora crassa GRASP NCU09217T0	169	PLRLYIYN
Schizosaccharomyces pombe GRASP SPAC1D4.02c	168	PLRLYIYN
Yarrowia lipolytica GRASP 68261	230	PIQLFVYN
Pichia pastoris GRASP 40255	209	TLVLYVYN

Barlow et al. Figure S5B

Homo sapiens GMAP210 NP_004230.2	1	--MSSWLG-------GLGSGLGQSLGQVGGSLASLTGQISNFTKDML
Rattus norvegicus GMAP210 NP_001178701.1	1	--MSSWLG-------GLGSGLGQSLGQVGGSLASLTGQISNFTKDML
Xenopus tropicalis GMAP210 450527	1	--MASWFG-------GLSSGIGQSLGQVGGSLSSITGQISNFTKDML
Gallus gallus GMAP210 XP_421324.3	1	--MASWLG-------GLGSGLGQSLGQVGGSLSSLTGQISSFTKDIL
Danio rerio GMAP210 NP_001265632.1	1	--MSSWLG-------GLGSGLGQSLGQVGGSLSSFTGQISNFTKDIL
Callorhinchus milii GMAP210 XP_007906720.1	1	--MASWFG-------GIGSGLGQSLGQVGGSLSSFTGQLSSFTKDIL
Petromyzon marinus GMAP210 ENSPMAP00000007865.1	1	--TMSWLG-----LGSLGGGLGQSLGQVGGSLSSLSGQISSLTKDLL
Botryllus schlosseri GMAP210 chr7.g44844.01.p	1	MMYGTWHS--------------------LTPIGHSSPQVHQVRRALL
Branchiostoma floridae GMAP210 113252	1	---
Nematostella vectensis GMAP210 107960	1	---MSWLG---------------------------------------
Amphimedon queenslandica GMAP210 2508048722	1	--MSSWFS--------------------GSSFTSLTDQITSFTKDVL
Trichoplax adhaerens GMAP210 18703	1	---MAWFT---------------------GSFNNITDQISNFTKDVL
Caenorhabditis elegans GMAP210 NP_001255174.1	1	--MSGWLR------------------NIQGQLTDLANEVLNEANDVL
Drosophila melanogaster GMAP210 FBpp0073859	1	---MSWLN---------------------SSLSQLKGQLTNLAQEVL
Hellobdella robusta GMAP210 191137	1	---MSWLQ---------------------TGLSSLTGQLGNFTREVL
Monosiga brevicollis GMAP210 MONBRDRAFT_09072T0	1	---MSWLS---------------------NSMTGLVGQVRSLTEDIL
Sphaeroforma arctica GMAP210 SARC_05656T0	1	---MSWFTDSLAKGLSDNLGASGITANLSNNFTNLTGQLSEFTNNLL
Rhizophagus irregularis GMAP210 3061	1	---
Mortierella verticillata GMAP210 MVEG_01046T0	1	---MSWLT---------------------KNVGSLGNLASRIASEAN
Cryptococcus neoformans GMAP210 2453	1	---
Ustilago maydis GMAP210 3240	1	---
Thecamonas trahens GMAP210 AMSG_02016T0	1	-------------------------------MGTYAEQAGELTKDLL
Dictyostelium discoideum GMAP210 DDB0346834	1	---
Dictyostelium fasciculatum GMAP210 DFA1571496	1	---
Arabidopsis thaliana GMAP210 AT3G61570.1	1	---------------------------MWSSIENMKANLHKIVLDVH
Physcomitrella patens GMAP210 Phpat.003G072700.1.p	1	---
Klebsormidium flaccidum GMAP210 kfl00170_0140	1	---------------------------MWSSIAKLKDNFAAIANDVL
Cryptosporidium parvum GMAP210 cgd3_3310	1	---------------------------MRAAWGALKGAVQELNDALL
Thalassiosira pseudonana GMAP210 9503	1	-------------------MWGSLARGITQSVKDTAGAVAPHAGDVL

Barlow et al. Figure S5C

```
Homo sapiens GMAP210 NP_004230.2                      1757  RQEMLDDVQKKLMSLANSSEGKVDKVLMRNLFIGHFHTPKN--QRHEVLRLMGSILGVRR
Danio rerio GMAP210 NP_001265632.1                    1761  RQEMLEEAQRKLMNLLNTTEGKVDKVLMKNLYLGYFHTPQN--KRGEVLRLMGNVLGLDR
Drosophila melanogaster GMAP210 FBpp0073859           1195  LKEANGQLEQRLSSSESSQTDKIDKSLIKSLLIGYVVSGHA-GDKQQVLRMISSVLDFNA
Amphimedon queenslandica GMAP210 2508048722           800   YQLTLKKCRAKIEQLSCDQDTNVDKQLVRNLFLSYISKQDEIKKRDKIVHVLQKVLDIDQ
Saccharomyces cerevisiae GMAP210(Sgm1p) NP_014859.3   386   LNEHLTKALAMLKK--SSDSESVDKELISNLLISFVSIPRADPRKFEVLELLSNFLNWDE
Cryptococcus neoformans GMAP210 2453                  343   SNEHLKEALRRLRK--NQSDNNVDRRLVTNILLSFLMTSRGDPKRFEMLSLLATILSWDD
Mortierella verticillata GMAP210 MVEG_01046T0         834   QQGHLTEAMRRLKE--ENSQNTVDIPLISNLFISFLNIPRGDQKRFEILQLISGVLKFTD
Ustilago maydis GMAP210 3240                          316   LNEHLTEALRRLRN--DQSDSNVDKRLVTNLLISFLTTPRTDGKRYEMLNLIAGVLGWKE
Thecamonas trahens GMAP210 AMSG_02016T0               853   IKASMQKMMAAQQAL-TASDARIDKALVANLLVTYFTKADA--EKAGVLSVLSSMLDLDE
Dictyostelium discoideum GMAP210 DDB0346834           519   LKVAFDKTILRLGDMCLQEQESVDKRVVSKLFLNYFSGN----KKTEILELIAKILNFSD
Arabidopsis thaliana GMAP210 AT3G61570.1              544   VRRVLEQSMTRLNRMSMESDYLVDRRIVIKLLVTYFQKNHN----KEVLDLMVRMLGFSE
Klebsormidium flaccidum GMAP210 kfl00170_0140         662   LRKALEQSMTRIHRMSSDSDQYVDRRIVIKLLVTYFEKRHS----AEALDLMARILGFTE
Thalassiosira pseudonana GMAP210 9503                 828   LRRALDEAISRLQ---TSQEDVIDRSLIKNIILDWHAKKGK--AKQDVMILLGSILHFTE

Homo sapiens GMAP210 NP_004230.2                      1815  EEMEQLFHDDQGG-------VTRWMTGWL-GG
Danio rerio GMAP210 NP_001265632.1                    1819  DEVGQLLKEEVKSG------MTGWVSSWLGGR
Drosophila melanogaster GMAP210 FBpp0073859           1254  QEADKVGLNKQ-------------QSSW--LG
Amphimedon queenslandica GMAP210 2508048722           860   SEIEKV-LGRK--------------AMW--LP
Saccharomyces cerevisiae GMAP210(Sgm1p) NP_014859.3   444   DKKQQAGLISN---------------------
Cryptococcus neoformans GMAP210 2453                  401   AEREKAGLQRQ--------------GAVGGGG
Mortierella verticillata GMAP210 MVEG_01046T0         892   EQREQAGLIRKAGG----------------LG
Ustilago maydis GMAP210 3240                          374   EERELAGLQKSAGA------VRASMGSSRGSA
Thecamonas trahens GMAP210 AMSG_02016T0               910   EQQAAIGVGAGAG-------LTGWISSW--FG
Dictyostelium discoideum GMAP210 DDB0346834           575   AEKISIGLTKK--------------GQWSLLP
Arabidopsis thaliana GMAP210 AT3G61570.1              600   EDKERIGAAKQGGGKGVVRGVLGFPGRF--VG
Klebsormidium flaccidum GMAP210 kfl00170_0140         718   EDKKRVGLAQL-GRRGVVSGVLGAPGRI--VG
Thalassiosira pseudonana GMAP210 9503                 883   DEKDKAFISEGPGT----------------VD
```

Barlow et al. Figure S5D

```
Homo sapiens Golgin-84 NP_005104.3                673   VRKAASSIDQFSIRLGIFLRRYPIARVFVIIYMALLHLWVMIVLLTYT---
Danio rerio Golgin-84 NP_998576.1                 702   VRKAASTIDRFSIRLGIFLRRYPMARVFVIIYMALLHLWVMIVLLTYT---
Callorhinchus milii Golgin-84 XP_007902048.1      680   VRKAANTIDRFSIRLGIFLRRYPIARIFVIIYMVLLHLWVMIVLLTYT---
Ciona intestinalis Golgin-84 239131               680   VRKAASVLDKFSIRLGIFLKRYPPARLFVLIYMGLLHVWVMIVLLTYS---
Amphimedon queenslandica Golgin-84 2508051726     712   VRNTLDSVDKIGLRVAWVLRRYPCVRLLTIGYILLLHLWVMIVLFTYQ---
Salpingoeca rosetta Golgin-84 PTSG_02257T0        718   FKTAANALDAISIRLGVFLRRYPAARILVIIYMILLHLWVMIVLLTTHHVP
Capsaspora owczarzaki Golgin-84 CAOG_09625T0      446   VRQAATVLDTFSIRLGRFLRIYPMARVFVIFYMLLLHLWVMIVLFTYS---
Acanthamoeba castellanii Golgin-84 ELR13889.1     471   TLRAASALDNFTAQVGRILRRNPLARLLLILYMVFLHVWVLFLLSRP----
Bigelowiella natans Golgin-84 75024              1030   VAKAMRFLDSFSSFISVMLRRNPAARLFFGLYVLMIHLWVLFVLLHFMGHE
Phytophthora sojae Golgin-84 297683               519   VVTALDVLDRWLLFLGRVFVQAPFARLGMLCYVVLIHFWVFMILSFHT---
Arabidopsis thaliana Golgin-84 AT1G18190.1        596   LGWLVMQLNAIFISGTVFLRRNPTAKIWAVVYLVCLHLWVLYILLSH----
Bodo saltans Golgin-84 BS19790                    473   VANAAQGLDKVSLKTGSFLRRNALLRVMLVAYVMMLHVWVFLVLTM-----
Naegleria gruberi Golgin-84 69983                 603   VAHVMAFIDSFSISTGSILRRLPLLRILLVIYIITLHIWVFYILSHL----
Naegleria gruberi CASP 30519                      526   RLNNMNAAEKLIYRCSGLMLSHKLGRLFLFFYLVTLHTLVFFTLSKLTGLT
Bodo saltans CASP BS41845                         595   TVRR--AADGVAVLVANLVVHSPTTRLFLVGYLIALHAIVMITTYVMAFRG
Chrysochromulina tobin CASP KOO27568.1            437   RYAELPAPEKLMLNFSQFFLANRHARLFLFGYMVCLHLLVSGAMYAASHHC
Bigelowiella natans CASP 73975                    715   HYDNLSTQEKIIHKFWEFLFSKHSHRFLLFGYALFLHFLVFVTLWRHTHIP
Phytophthora sojae CASP 484998                    659   RFTNLNPVDKILLTSAKLLLAHRITRNMAFGYMLLLHFLVVATLYSFMHVC
Toxoplasma gondii CASP TGME49_278890             1032   RLQTLSGAERIVVIWGRLLLSCRATRLFALFYFVLLHFLVFLVLFYHADLQ
Guillardia theta CASP 106475                      639   RKEKMPLHERLMLEMSQFFLGNSVARKFLFFYMVIMHILVFVTLYRFTHNT
Arabidopsis thaliana CASP AT3G18480.1             617   RIKDLGIRDRITLSSGRFLLGNKYARTFAFFYTIGLHVLVFTCLYRMSAYS
Acanthamoeba castellanii CASP ELR24051.1          553   RYKGLNTAEKVTLHTGRFFLANKYSRTFIFFYALALHLLVFLTLYKLANTA
Batrachochytrium dendrobatidis CASP 88000         597   RFHSMNPAERAALSLTRLLSTNKYSRWIFVIYSGALHLLVFFTLFQLSMSD
Saccharomyces cerevisiae CASP NP_012742.1         588   KNKKLSALEKLFSSFAKVILQNKMTRMVFLFYCIGLHGLVFMMSMYVINIS
Capsaspora owczarzaki CASP CAOG_05174T0           694   KVQALNATERITLSMGRFIMSNKYARSFVFFYSIILHALVFLVLYKLSWSQ
Salpingoeca rosetta CASP PTSG_00349T0             631   RIMNLNAGDRLTLTLGKAVMGSKNARLIFLGYAIIVHLLIFVVLYKYSHAG
Amphimedon queenslandica CASP 2508059134          570   KYMQLSGPDKATLILGRFILSNKVARMITFFYIIIIHLIIMLVLAKLVNTA
Nematostella vectensis CASP 40279                 381   RYMNLGPHEKVTLNLGRFVLGSKMARTIAFFYMLFLHCLVFLV--------
Ciona intestinalis CASP 286365                    581   KYMNLTPPEKVTLGLGKVILSSKIARNIFFFYMLFMHCLLYIVLYKYA-YA
Danio rerio CASP NP_001003514.1                   593   RYQSLSPWDKATLSLGRVILSNKTARTIAFFYTLMLHCLVFLVLYKAA-WS
Homo sapiens CASP XP_005250211.1                  682   KYLSLSPWDKATLSMGRLVLSNKMARTIGFFYTLFLHCLVFLVLYKLA-WS
```

Barlow et al. Figure S5E

Homo sapiens Golgin-84 NP_005104.3	1	-MSWF---VDLAGKAEDLLNRVDQGA
Rattus norvegicus Golgin-84 NP_001028237.1	1	-MSWF---ADLAGRAEDLLNRVDQGA
Xenopus tropicalis Golgin-84 475595	1	-MSWF---TGLAGRAEDFLNLVDQGA
Gallus gallus Golgin-84 XP_421329.2	1	-MSWL---ADLAGKAEDLLNRVDQGA
Danio rerio Golgin-84 NP_998576.1	1	-MSWF---VDLAGKAEDFLNKVDQGA
Callorhinchus milii Golgin-84 XP_007902048.1	1	-MSWF---TDLAGKAEDFLNKVDQGA
Ciona intestinalis Golgin-84 239131	1	-MSWL---NNLAGKAESLLNNIDQSA
Branchiostoma floridae Golgin-84 154839	1	-MSWI---SDFAGKAEALLNKVDQTA
Drosophila melanogaster Golgin-84 FBpp0084013	1	MSSWI---TGLADKAENILNKLDQNA
Caenorhabditis elegans Golgin-84 NP_492137.1	1	-MSWLSKVSDIAGAAENLLNKLDEKT
Nematostella vectensis Golgin-84 239710	1	-MSWF---SELAVKAESLLEKVDNTA
Amphimedon queenslandica Golgin-84 2508051726	1	-MAWF---SSIAGKAEQLLNQLDEAA
Mnemiopsis leidyi Golgin-84 ML14421a	1	MAGLV---------------------
Monosiga brevicollis Golgin-84 MONBRDRAFT_23784T0	1	MASLF-------TSLTMMLEDADRRA
Salpingoeca rosetta Golgin-84 PTSG_02257T0	1	MSNWF---AEFRDTAVSVLEQADQLA
Capsaspora owczarzaki Golgin-84 CAOG_09625T0	1	--------------------------
Sphaeroforma arctica Golgin-84 SARC_09713T0	1	-MNLL-------------LQPPNVAS
Acanthamoeba castellanii Golgin-84 ELR13889.1	1	--------------------------
Thecamonas trahens Golgin-84 AMSG_05059T0	1	MSSWF-------STASSFLDAMDQSA
Arabidopsis thaliana Golgin-84 AT1G18190.1	1	MANWI---SSKLKAAETILQQLDQQA
Arabidopsis thaliana Golgin-84 AT2G19950.2	1	MASWL-------KAAEDLFEVVDRRA
Physcomitrella patens Golgin-84 Phpat.002G025400.2.p	1	MAGWL---SSRLKAAEQLLQQIDQQA
Klebsormidium flaccidum Golgin-84 kfl00053_0020	1	MASWL---QNQFKKAEELFEAVDQTA
Klebsormidium flaccidum Golgin-84 kfl00291_0020	1	MASWL---SSRLKAAEQFLNQIDATA
Volvox carteri Golgin-84 Vocar20014314m	1	MASWI---TAQLKAAEGLLEAVDKTV
Bigelowiella natans Golgin-84 75024	1	MSNWI-------EWASKALDDLDSGT
Reticulomyxa filosa Golgin-84 ETO03839	1	--------------------------
Thalassiosira pseudonana Golgin-84 21843	1	MSRWL-------RNVNALLENLDSQV
Phytophthora sojae Golgin-84 297683	1	-MNWV---SSSLELAGSLLESVDQQA
Bodo saltans Golgin-84 BS19790	1	-MSFL-------QGVTSFLESVDQKA
Naegleria gruberi Golgin-84 69983	1	--MWA---NSVLKWAEQTLETVDRST

Barlow et al. Figure S5F

```
Homo sapiens TMF NP_009045.2                        985   IIENLQSQLKLREGEITHLQLEIGNLEKTRSIMAEELVKLTNQNDELEEKVKEIPKLRTQ
Danio rerio TMF NP_001003522.1                      1028  VIESLQSQLKLREGEITQLQMEIASLERSRTVMSEELVRLTNENDEMESKVKEIPRLKVQ
Callorhinchus milii TMF XP_007893394.1              1029  VFENLQSQLKQREGEIAQLQLEISSLEKTRAIMAEELVKLTTQNDELQEKVNEIPILREQ
Ciona intestinalis TMF 293917                       779   LLENLQSQLKQRDGEIAQLQGEINTLERTRSSMAEEIVRLTNENEEMEVTVGQVDELTRK
Drosophila melanogaster TMF FBpp0070954             816   TFEHLQALLKQRDGELTHLQWEVSRLQAERSVLDAEISNLTIELETMKEKQQMYEVMEKG
Amphimedon queenslandica TMF 2508061876             937   VLEQLQSSLKQKEGELSNSQLMVASLERSRASLTQELATVSERNEVLEQKVKMIPDLQQK
Saccharomyces cerevisiae TMF(Sgm1p) NP_012668.3     597   LVNKLSTELKRLEGELSASKELYDNLLKEKTKANDEILRLLEENDKFNEVNKQKDDLLKR
Batrachochytrium dendrobatidis TMF 87032            1405  IIERLQLMIKQSESQISSLQLQLRMATQTRNEMSDEFVRLTAESEELKAKADKAQAIESE
Thecamonas trahens TMF AMSG_08612T2                 736   ALETMLARAKLQGDEIEALKRHMSSLSQTRQFLEDRLVESNASAEALAERVHQLEAFAGR
Dictyostelium discoideum TMF DDB0237556              972   SLEYLQSSLSQKEGEAMSLQAQVQSLNHSRKKLEDELVKLTTDNEELLSECKELKLQRQE
Mastigamoeba balamuthi TMF m51a1_g1405              605   -----------QRSELTALQEQISVLKSARADLEEEIVRISTRNTELQQEIERLKRAGQE
Arabidopsis thaliana TMF AT1G79830.1                849   TPSAYEATLRQKEGELASYMTRLASMESIRDSLAEELVKMTAECEKLRGEADRVPGIKAE
Phytophthora sojae TMF 497264                       16    GLSQLQQRLRLREGENRMLKQQLEALEARQKQTTDEIVRLSTRNALLESGEAQREQAQQE
Ectocarpus siliculosus TMF Esi0075_0002             1278  EVERLHSLAKQRQGQADVLQQRLEAVQATRDALTEEVTSLGRRNTELEALAKAVPLLRDQ
Naegleria gruberi TMF 79996                         542   TNSELLEIIEKKNNKIKTLFVQIRDLEEIKEDLSERLAMYKQTNQTLANEINEKELTLKQ

Homo sapiens TMF NP_009045.2                        1045  LRDLDQRYNTILQMYGEKAEEAEELRLDLEDVKNMYKTQIDELLRQSLS--
Danio rerio TMF NP_001003522.1                      1088  LKDLEQRHNTILQMYGEKAEEAEELRLDLEDVKNMYKLQIDELLKNQKQ--
Callorhinchus milii TMF XP_007893394.1              1089  LKDIDQRYNTILQMYGEKAEEAEELRLDLEDVKNMYKAQIDELLKQRPH--
Ciona intestinalis TMF 293917                       839   LKEVTARHDAVLTMYGEKAEEAEELKLDLEDVKTMYRNQTQHAKISIVTVQ
Drosophila melanogaster TMF FBpp0070954             876   YEDLQHRYDALLQMYGEKVERTEELELDLTELKAAYKLQIDELLAAPPPNL
Amphimedon queenslandica TMF 2508061876             997   LKEMSQKHEALLQMFGEKAEETEELRMDIEDLKTMYRQQIEDLISKNQQHK
Saccharomyces cerevisiae TMF(Sgm1p) NP_012668.3     657   VEQMQSKLETSLQLLGEKTEQVEELENDVSDLKEMMHQQVQQMVEMQGKMR
Batrachochytrium dendrobatidis TMF 87032            1465  LTELQKRYNTALEILGEKTERAEELQEDIKDMRLAFKAQVEDLVRQLGRHA
Thecamonas trahens TMF AMSG_08612T2                 796   TAEIERRLNAALEIIGEKEETIEDIRADRDLYKQMYKEQLPGLVEQIELQR
Dictyostelium discoideum TMF DDB0237556              1032  IKDIQQRYQTTLLMLGEKEEAVNELRLDIMDLKDLYKNQINELLLQIEVLK
Mastigamoeba balamuthi TMF m51a1_g1405              655   AEEAERRHQAALVLLGEREEQLAELRLDVEDLKETYRMQLNSLALQLEHNG
Arabidopsis thaliana TMF AT1G79830.1                909   LEALRQRHAAALELMGERDEELEELRADIVDLKEMYREQVNMLVNKIQ---
Phytophthora sojae TMF 497264                       76    LAQLQKHQVVLLELFGEKEEQVEELQAEVSELKAFYRKQLDTLATHNEQQQ
Ectocarpus siliculosus TMF Esi0075_0002             1338  ALELQDKNGVLLDLLGEKTEDLEAVQADMREMQNMYRAQYDELLTRAGAGV
Naegleria gruberi TMF 79996                         602   FNDLKFRYEAALDLIGEKEEALQTIQEEFKYVKETFRTQISSLLKEIETLK
```

Barlow et al. Figure S5G
